# A review on clinical cases of rare mold infections and their antifungal treatment strategies

**DOI:** 10.3389/fmicb.2026.1903380

**Published:** 2026-09-11

**Authors:** Kaile Zhai, Yuhua Chang, Lin Liu, Ximeng Duan, Shuqin Wang, Xiuyun Li

**Affiliations:** 1 Infection and Microbiology Research Laboratory for Women and Children, Shandong Provincial Maternal and Child Health Care Hospital·Affiliated to Qingdao·University, Jinan, China; 2 School of Pharmacy, Qingdao University, Qingdao, China; 3 Gynecology Department, Shandong Provincial Maternal and Child Health Care Hospital Affiliated to Qingdao University, Jinan, China; 4 Pediatric Department, Shandong Provincial Maternal and Child Health Care Hospital Affiliated to Qingdao University, Jinan, China; 5 Department of Pediatric Urology, Shandong Provincial Hospital Affiliated to Shandong First Medical University, Jinan, China

**Keywords:** antifungal susceptibility testing, antifungal treatment strategies, identification techniques, mold infection cases, natural products, rare molds, susceptible populations

## Abstract

In recent years, with the growing population of immunocompromised individuals, infections caused by rare environmental molds have become increasingly prevalent and clinically significant. Due to frequent misdiagnosis and difficult clinical management, the overall disease burden of such infections poses a major challenge in clinical practice. This article aims to fill the knowledge gap by reviewing cases of rare mold infections from January 2020 to February 2026, analyzing the susceptible populations, identification techniques, antifungal susceptibility testing, and antifungal treatment strategies. By integrating clinical case data, we found that although most rare mold infections respond well to conventional antifungal therapy, a small number of cases still result in death. This phenomenon is attributed to the rarity of pathogens, the lack of recommended antifungal agents, or unrecognized intrinsic drug resistance, which poses a serious threat to clinical management and public health. Therefore, we further summarize research on natural products against these rare molds, aiming to provide new therapeutic insights for the management of these challenging infections. The significance of this review lies in systematically synthesizing fragmented case evidence into consolidated knowledge. By deeply integrating clinical information with rare mold identification, we constructed a comprehensive strategic framework covering rapid identification, targeted treatment, and the exploration of potential therapeutic agents. This provides evidence-based guidance for the future development of standardized diagnosis and treatment protocols for such rare mold infections.

## Graphical Abstract

**Figure:**
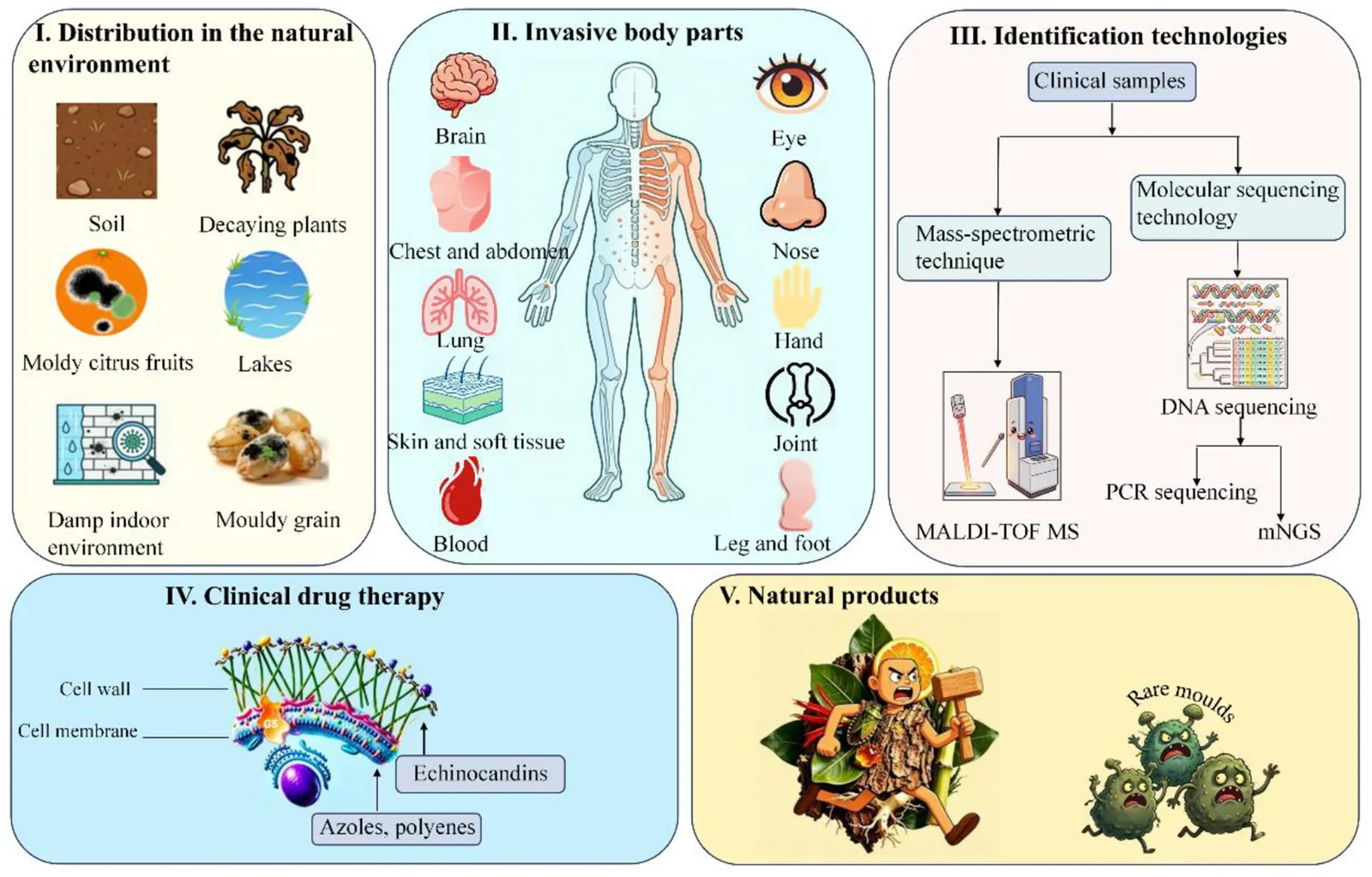
The journey of rare moulds, from the natural environment to clinical treatment.

## 1 Introduction

In recent years, the global burden of fungal infections has been on a steady rise, posing a substantial public health threat worldwide (Fang et al., 2023). Globally, an estimated 3.75 million deaths are attributed to fungal infections annually (Ikuta et al., 2024). Moreover, the COVID-19 pandemic has markedly increased the susceptibility to invasive fungal diseases (Hoenigl et al., 2022). Notably, molds have emerged as a critical group of opportunistic fungal pathogens in clinical practice. Although invasive aspergillosis and mucormycosis are the most common invasive mold infections, the number of cases of infections caused by rare molds, including penicilliosis and fusariosis among others, has also increased (Park et al., 2011). The reason for the increase in cases of rare mold infections remains unclear, and this trend may be associated with the widespread use of immunosuppressive agents, global population migration, and altered mold ecological environments driven by global warming (Konkel Neabore, 2024; Ashraf et al., 2020).

Most rare molds present atypical clinical features and are difficult to detect via routine microbiological examinations. Meanwhile, uniform detection standards and antifungal susceptibility breakpoints for these rare molds are still unavailable, collectively rendering them a severe threat to human health (Lackner et al., 2012). Additionally, some rare molds have intrinsic or acquired resistance to existing conventional antifungal agents, which further complicates the clinical treatment of these infections (Boutin and Luong, 2024). Clinically available antifungal agents primarily consist of three major classes: polyenes, azoles, and echinocandins (Anderson et al., 2014; Odds et al., 2003; Denning, 2003). However, the existing antifungal agents still have limitations in their antifungal spectrum, failing to cover these rare molds. Moreover, some antifungal agents have significant adverse reactions, such as visual impairment caused by voriconazole and renal toxicity caused by amphotericin B (Patterson et al., 2016). Given the scarcity and insufficiency of existing antifungal agents, it is of great significance to explore novel and highly effective antifungal agents derived from natural products in order to overcome rare mold infections (Argüelles et al., 2024).

To deepen clinicians' understanding of rare mold infections, enhance awareness of early diagnosis, and provide references for optimizing individualized treatment, this review summarizes case reports of rare mold infections with no more than three cases published between January 2020 and February 2026. We conducted the literature search using PubMed as the primary database. The search strategy consisted of two main components: a. for mold species already listed in the global guideline for the diagnosis and management of rare mold infections (e.g., the genera/species discussed in Sections 2.1, 2.2, 2.4, 2.5, and 2.7), we searched using the keywords [Genus species] AND case reports; b. for mold species not included in the above guideline, we searched using the keywords mold AND case reports. All searches were limited to publications from January 2020 to February 2026. From the retrieved results, we selected only those case reports describing no more than three cases of rare mold infections. This approach ensured that our review focused on truly uncommon infections while maintaining consistency with the case-count-based organizational logic of the manuscript. Based on the included cases, we summarized the susceptible populations, identification techniques, antifungal susceptibility testing, antifungal treatment strategies, and explored potential therapeutic options for these rare mold infections based on natural products.

## 2 Rare molds

### 2.1 *Penicillium spp*.

*Penicillium spp*., which are widely present in the natural environment, are usually considered laboratory contaminants and rarely cause human diseases (Visagie et al., 2014; Hoenigl et al., 2021; Atalay et al., 2016). *Penicillium spp*. have a dual nature. On the one hand, the well-known *Penicillium chrysogenum* is the producer of the antibiotic penicillin, saving countless lives (Barreiro and García-Estrada, 2019). On the other hand, as toxin-producing molds, they compromise food safety and storage and pose potential health risks to immunocompromised individuals. With the advancement of medical technology and fungal identification techniques, some rare pathogenic molds, such as *Penicillium citrinum* and *P. chrysogenum*, have been found to infect humans (Hesse et al., 2017; De Oliveira et al., 2023). Currently, the list of rare pathogens within *Penicillium spp*. is continually being updated. This trend does not signify that the pathogenicity of these rare molds has increased, but seemingly instead points to advances in our diagnostic capabilities alongside the growing population of immunocompromised patients (Mehta et al., 2021).

**Figure 1:**
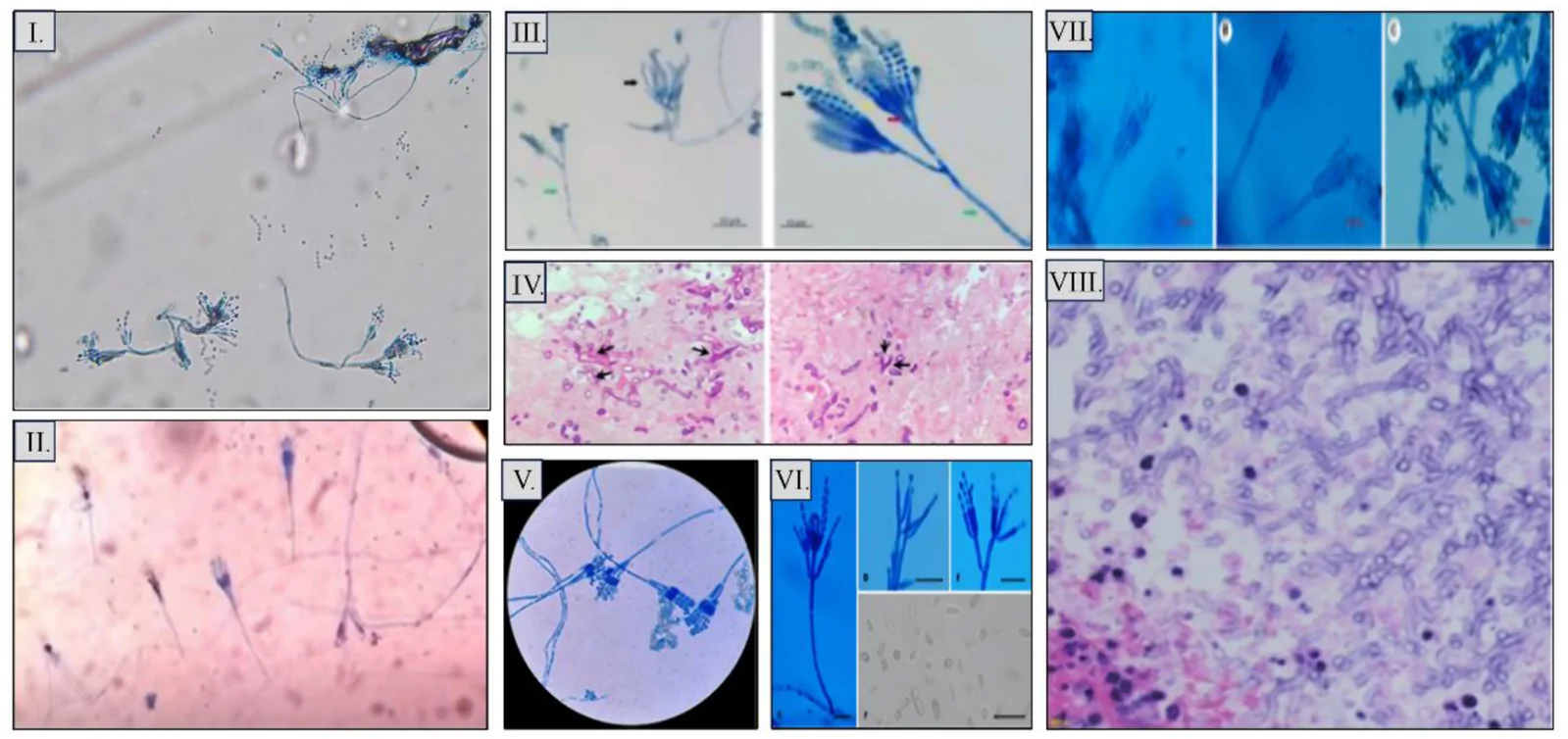
Morphological images of species in the *Penicillium spp*. **(I)** Microscopic morphology of *P. citrinum* in a sputum specimen (lactophenol cotton blue staining) (Zhao et al., 2020). **(II)**. Microscopic morphology of *P. citrinum* in a sputum specimen (lactophenol cotton blue staining) (Beena et al., 2021). Reprinted from Gupta, P., Thakur, A., Rudramurthy, S. M., Gupta, A., Ghosh, A., Kaur, H., et al. (2022). Initial case report of cladorrhinum samala Mycotic Keratitis. *Cornea* 41, 1302–1304. doi: 10.1097/ICO.0000000000002992, with permission from Elsevier. **(III)** Microscopic morphology of *P. chrysogenum* in a meningeal specimen (lactophenol cotton blue staining) (De Oliveira et al., 2023). Adapted from Hirao, Y., Morioka, H., Ozaki, S., Kawachi, M., Ban, S., Yaguchi, T., et al. (2024). A case of invasive rhinosinusitis caused by Penicillium brasilianum. *Med. Mycol. J*. 65, 111–115. doi: 10.3314/mmj.24-00015, with permission from the Japanese Society for Medical Mycology. **(IV)** Microscopic morphology of *P. chrysogenum* in a sinus specimen (Bhavsar et al., 2022). Reprinted from Gupta, P., Thakur, A., Rudramurthy, S. M., Gupta, A., Ghosh, A., Kaur, H., et al. (2022). Initial case report of cladorrhinum samala Mycotic Keratitis. *Cornea* 41, 1302–1304. doi: 10.1097/ICO.0000000000002992, with permission from Elsevier. **(V)** Microscopic morphology of *P. oxalicum* in a lung specimen (lactophenol cotton blue staining) (Wang et al., 2024). **(VI)** Microscopic morphology of *P. digitatum* in a lung specimen (lactophenol cotton blue staining) (Iturrieta-González et al., 2022). **(VII)** Microscopic morphology of *P. digitatum* in a lung specimen (lactophenol cotton blue staining) (Shi et al., 2024). **(VIII)** Microscopic morphology of *P. brasilinum* in a paranasal sinus specimen (giemsa staining).

*P. citrinum* is a well-known mold that is ubiquitous in the natural environment, such as citrus fruits, tropical spices, and cereals. It can occasionally cause human infections (Mok et al., 1997). Based on a comprehensive literature search, human infections caused by *P. citrinum* had been reported only four times prior to 2020. Among these cases, one was keratitis, two were pneumonia, and the remaining one presented with skin lesions (Hesse et al., 2017). From January 2020 to February 2026, two cases of human infections caused by *P. citrinum* have been reported. In 2020, a fungal pathogen isolated from sputum samples of a patient with diabetes mellitus was sent to a reference laboratory for identification and confirmed as *P. citrinum* by polymerase chain reaction (PCR) sequencing. The isolate was susceptible to amphotericin B but resistant to voriconazole. Following antifungal therapy with amphotericin B, the patient's lesions in the chest gradually regressed and resolved completely within 4 months (Figure 1I) (Zhao et al., 2020). In 2021, *P. citrinum* was identified by deoxyribonucleic acid (DNA) sequencing in the lungs of a patient with multiple myeloma, whose pulmonary infection improved after 2 weeks of intravenous liposomal amphotericin B therapy (Figure 1II) (Beena et al., 2021). The above cases indicate that when *P. citrinum* is isolated from clinical samples, especially respiratory samples, of immunocompromised patients, it should be regarded as a potential pathogen rather than simply discarded as a contaminating mold. In both cases mentioned above, infections caused by *P. citrinum* responded significantly to amphotericin B, which suggests that amphotericin B can be considered the core drug for initial treatment when the infection is confirmed or highly suspected.

*P. chrysogenum* is a widely distributed fungus found in diverse habitats worldwide, including extreme temperature environments such as Antarctic soil (De Sousa et al., 2017). It is also an important microorganism necessary for the production of penicillin. Before 2020, 13 cases of human infections caused by *P. chrysogenum* had been reported, which were predominantly observed in sites such as the lungs, eyes, skin, and intestinal tract, with the majority occurring in immunocompromised individuals. From January 2020 to February 2026, only two cases of *P. chrysogenum*-related infections have been reported. In 2022, this mold was first identified in the brain of an immunocompetent female patient with frontal headaches, with pathogen confirmation accomplished via internal transcribed spacer (MIC) and β-tubulin gene sequencing. The patient received intravenous voriconazole combined with amphotericin B and intracranial pressure reduction therapy. However, the mold infection continued to progress, and the patient ultimately died of mixed shock (Figure 1III) (De Oliveira et al., 2023). In this case, two molecular identification methods were used in combination for pathogen detection, enabling mutual verification and greatly improving the diagnostic accuracy of rare mold species. In 2022, *P. chrysogenum* was detected by PCR assay in formalin-fixed tissue samples from the nasal cavity of a male patient with T-cell acute lymphoblastic leukemia, after the samples were sent to the New York State Department of Health Wadsworth Center for identification. The patient was treated with amphotericin B for 8 weeks. After the condition stabilized, he received posaconazole maintenance therapy and underwent surgical debridement. After 12 months of follow-up, sinusitis did not recur (Figure 1IV) (Bhavsar et al., 2022). These cases indicate that *P. chrysogenum* is an opportunistic pathogenic mold that primarily threatens immunocompromised individuals, and restoring the patient's immune function is essential for infection control. In rare instances, *P. chrysogenum* can also affect immunocompetent hosts, as exemplified by the first reported case, which remains extremely uncommon. Although *P. chrysogenum* infection is uncommon and presents significant treatment challenges, aggressive surgical intervention combined with long-term sequential antifungal therapy may achieve a favorable outcome.

*Penicillium oxalicum* is a common fungus widely found in soil and has relatively low pathogenicity to humans. According to literature searches, no human infections caused by *P. oxalicum* were reported prior to 2020, and only one case has been documented from January 2020 to February 2026. In 2024, *P. oxalicum* and *Penicillium variotii* were detected in the lungs of a 56-year-old female with poorly controlled type 2 diabetes and hypertension, via bronchoalveolar lavage fluid (BALF) culture and ITS sequencing. *P. oxalicum* is susceptible to both voriconazole and amphotericin B. *P. variotii* is resistant to voriconazole but susceptible to amphotericin B. After the patient used amphotericin B cholesterol sulfate, her pleural effusion was completely absorbed, her pneumonia symptoms had significantly improved, and she was discharged (Figure 1V) (Wang et al., 2024). This is the first reported case of pneumonia and pleural effusion caused jointly by *P. oxalicum* and *P. variotii* worldwide. Most opportunistic fungal pneumonias are caused by a single pathogen, whereas mixed infections are particularly rare. For suspected *P. oxalicum* infection, amphotericin B should be prioritized for empirical therapy based on antifungal susceptibility testing results of both the isolate and co-existing pathogens.

*Penicillium digitatum* is a common plant pathogen that mainly causes rotting of citrus fruits and rarely causes human infections. Before 2020, only one case of lung infection caused by *P. digitatum* had been reported worldwide (Oshikata et al., 2013). From January 2020 to February 2026, three cases of human infections caused by *P. digitatum* have been reported. In 2022, *P. digitatum* was detected in the lungs of a 20-year-old pregnant woman via β-tubulin gene sequencing, and the isolate was susceptible to amphotericin B, voriconazole, and itraconazole. This patient showed rapid improvement and recovered after monotherapy with itraconazole (Figure 1VI) (Iturrieta-González et al., 2022). In 2024, *P. digitatum* was identified in the lungs of a patient with emphysema via metagenomic next-generation sequencing (mNGS), and the isolate was susceptible to voriconazole, itraconazole, micafungin, and amphotericin B. After 1 month of oral itraconazole treatment, the pulmonary infection was effectively controlled, and no recurrence of infection was observed during 1 year of follow-up (Figure 1VII) (Shi et al., 2024). In 2026, *P. digitatum* was detected via mNGS in the lungs of a lymphoma patient. After 10 days of treatment with voriconazole, the fever resolved, but the patient eventually died of multiple organ failure (Chen et al., 2026). The above cases indicate that *P. digitatum* infection mainly affects the lungs and is more common in immunocompromised patients. The drug susceptibility of *P. digitatum* may vary among different populations or infection sites, but the available evidence supports the effectiveness of triazole antifungal agents. Furthermore, through molecular biological techniques, such as β-tubulin gene sequencing and mNGS, the precise identification of this mold can be achieved, thereby facilitating early diagnosis and enabling the timely initiation of long-term antifungal treatment, which is expected to successfully control such infections.

*Penicillium brasilianum* has been isolated from sources, including soil, plant endophytes, and onion bulbs (Bazioli et al., 2017; Cho et al., 2005; Fill et al., 2018). Its wide ecological distribution and ability to cause human diseases reflect its strong adaptability and pathogenic potential. According to available reports, no human infections caused by *P. brasilianum* were documented prior to 2020, and only one such case has been reported from January 2020 to February 2026. In 2024, invasive fungal rhinosinusitis due to *P. brasilianum* was diagnosed in a type 2 diabetic woman, with the pathogen confirmed by β-tubulin gene sequencing of sinus tissue. This isolate was susceptible to itraconazole, and the patient showed significant improvement in orbital apex syndrome following oral itraconazole solution combined with surgical intervention, but ultimately died due to heart failure (Figure 1VIII) (Hirao et al., 2024). This case highlights the significance of molecular identification techniques in accurately identifying molds. Although there were reports that matrix-assisted laser desorption/ionization time-of-flight mass spectrometry (MALDI-TOF MS) could identify *Penicillium spp*., due to the lack of *P. brasilianum* in the reference library, accurate identification of *P. brasilianum* could not be achieved through MALDI-TOF MS (Reeve et al., 2019). Eventually, the isolate was accurately identified as *P. brasilianum* via β-tubulin gene sequencing. Although the patient ultimately died of heart failure, the fact that the invasive fungal rhinosinusitis did not recur after antifungal therapy combined with endoscopic sinus surgery demonstrates that the successful management of *P. brasilianum* infection depends on multimodal comprehensive treatment.

In conclusion, *Penicillium spp*. are widely distributed in the natural environment. Although they rarely infect humans, they pose a fatal threat to immunocompromised individuals. *Penicillium spp*. present no specific clinical symptoms or imaging manifestations, so diagnosis usually requires molecular testing, such as mNGS, ITS sequencing, and β-tubulin gene sequencing. It is worth noting that DNA sequencing is the gold standard for fungal identification, with the β-tubulin gene serving as a particularly effective marker for species identification of *Penicillium spp*. (Visagie et al., 2014). Based on clinical case data collected from January 2020 to February 2026, infections caused by *Penicillium spp*. are more common in patients with meningitis, lymphoma, and diabetes. The global guideline for the diagnosis and management of rare mold infections recommends liposomal amphotericin B as the first-line treatment for *Penicillium spp*. infections (Hoenigl et al., 2021). However, our case analysis suggests that based on antifungal susceptibility testing, itraconazole can also be included in the antifungal treatment strategies for *Penicillium spp*. infections (Iturrieta-González et al., 2022; Shi et al., 2024).

### 2.2 *Fusarium spp*.

*Fusarium spp*. are molds widely distributed worldwide, particularly in tropical and subtropical regions, where they act predominantly as plant pathogens and are commonly found in plant debris, infected seeds, and soil (Hoenigl et al., 2021; Dean et al., 2012; Leslie and Summerell, 2006; Sharon and Shlezinger, 2013). However, occasionally they also found to be a kind of opportunistic pathogen for humans. In immunocompromised individuals, *Fusarium spp*. can cause severe disseminated infections, with the most common manifestations being sinusitis, pneumonia, or disseminated disease accompanied by fungemia and skin involvement (Nucci and Anaissie, 2007). In recent years, certain rare species of *Fusarium spp*. have been found to produce toxins that not only impair plant growth but also persist in grains and their products, causing diseases in humans and animals (Perochon and Doohan, 2024). With the widespread application of fungal identification techniques, more rare *Fusarium* species, such as *Fusarium dimerum, Fusarium falciformis* and *Fusarium verticillioides*, have been found to cause human infections as well (Risum et al., 2022; Larimore and Libertin, 2025; Feng et al., 2024).

**Figure 2:**
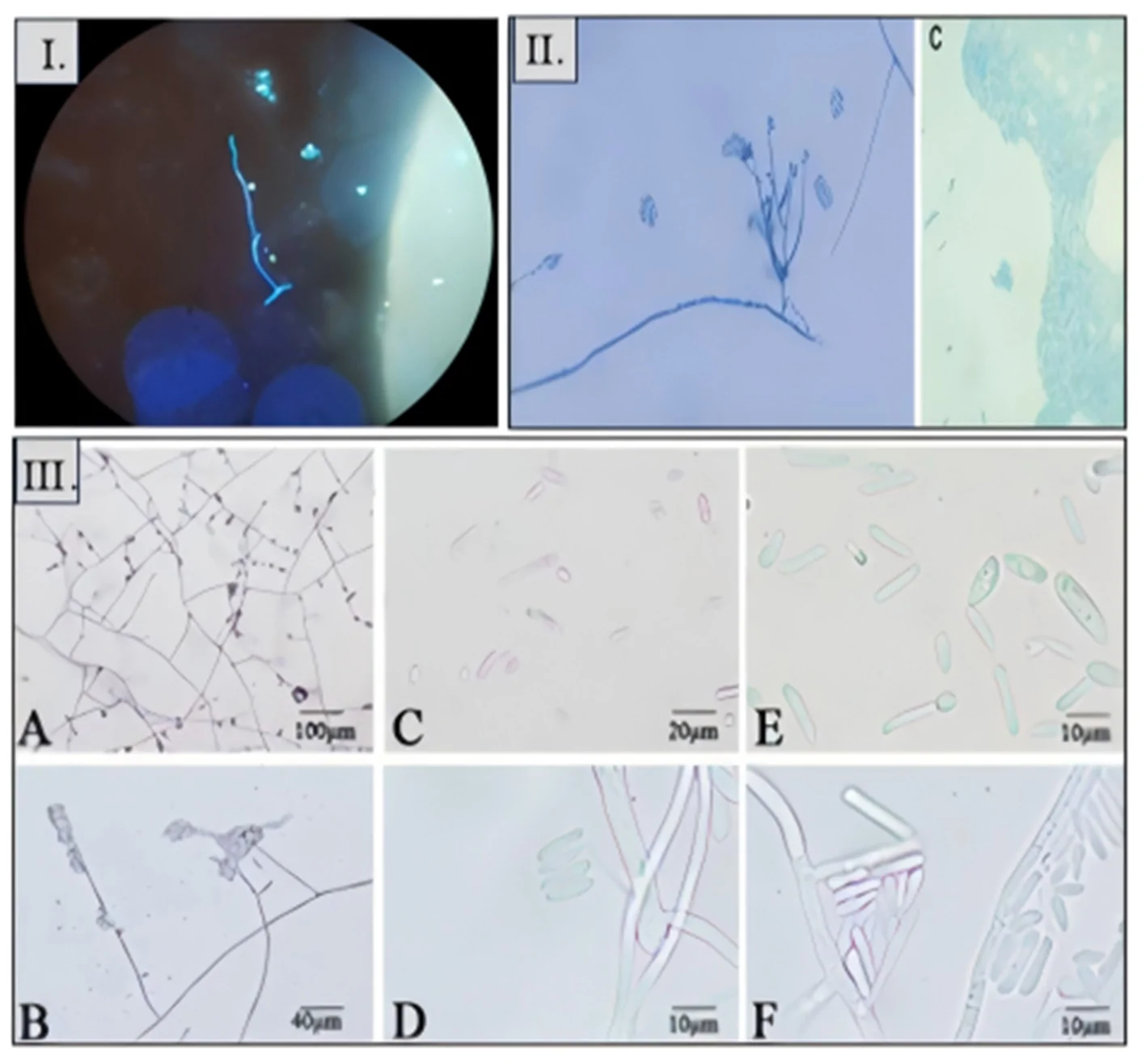
Morphological images of species in the *Fusarium spp*.**(I)** Microscopic morphology of *F. dimerum* in a paranasal sinus specimen (blankophor staining) (Risum et al., 2022). **(II)** Microscopic morphology of *F. verticillioides* in a blood specimen (lactophenol cotton blue staining) (Barberis et al., 2022). **(III)** Microscopic morphology of *F. verticillioides* in a foot specimen (Feng et al., 2024). Reprinted from Beena, H., Gupta, M., and Kindo, A. J. (2021). Pulmonary infection with Penicillium citrinum in a patient with multiple myeloma. *Indian J. Med. Microbiol*. 39, 259–261. doi: 10.1016/j.ijmmb.2021.03.001 and Criado, P. R., Belda Junior, W., and Da Matta, V. L. R. (2021). First report of cutaneous mycetoma by Paecilomyces variotii and the successful treatment with combined itraconazole and terbinafine along with resection surgeries. *Australas. J. Dermatol*. 62, e397–e399. doi: 10.1111/ajd.13592, with permission from Elsevier.

*F. dimerum* is a hyaline filamentous fungus that is commonly found in plants and soil. Before 2020, a total of 10 cases of *F. dimerum* infection were reported, mainly involving the eyes, heart, abdomen and skin. From January 2020 to February 2026, two cases of human infection caused by *F. dimerum* have been reported. In 2021, a patient with acute myeloid leukemia (AML) underwent central-line and peripheral blood cultures, and the results showed positive growth of *F. dimerum*. After 3 days of voriconazole treatment, the patient was switched to intravenous liposomal amphotericin B, leading to the resolution of the bloodstream infection (Alshaya et al., 2021). In 2022, *F. dimerum* was isolated from the paranasal sinuses of a 38-year-old female patient with short bowel syndrome and identified via ITS and 18S rDNA sequencing. This isolate was susceptible to amphotericin B but exhibits reduced *in vitro* susceptibility to azoles (i.e., voriconazole, posaconazole, and itraconazole). After treatment with voriconazole, the patient developed liver damage. After switching to liposomal amphotericin B, the disseminated fusariosis improved and the patient was discharged (Figure 2I) (Risum et al., 2022). In this case, ITS and 18S rDNA sequencing were jointly employed to identify *F. dimerum*. This approach overcomes the limitations of a single molecular marker and maximizes the accuracy and reliability of species identification for rare molds (De Filippis et al., 2017; Sakai et al., 2006). The above cases suggest that for severe and invasive *F. dimerum* infections, especially those accompanied by bloodstream infections, amphotericin B should be given priority as the initial treatment. If voriconazole is used, blood drug concentration monitoring must be conducted to optimize the therapeutic effect and reduce the risk of liver damage. Through active, persistent and continuously improved antifungal treatment strategies, complete cure of *F. dimerum* infection is possible.

*F. falciformis* is a soil-dwelling mold. No human infections caused by *F. falciformis* were reported before 2020, and two such cases have been reported from January 2020 to February 2026. In 2022, *F. falciformis* was isolated from the skin of a patient with acute myelogenous leukemia through combined morphology and DNA sequencing of the translation elongation factor 1-alpha (TEF) and RNA polymerase II second largest subunit (RPB2) genes at a reference laboratory. This isolate was resistant to voriconazole and posaconazole but susceptible to amphotericin B. After 5 months of treatment with liposomal amphotericin B, the mold infection partially improved; however, the patient transitioned to hospice care due to a relapse of acute myelogenous leukemia (Figure 2II) (Jabr et al., 2022). In 2025, *F. falciformis* was identified in the cornea of a patient with rheumatoid arthritis on adalimumab therapy, based on intraoperative cultures obtained during keratoplasty. This isolate demonstrated resistance to voriconazole and posaconazole and susceptibility to amphotericin B. After systemic therapy with intravenous liposomal amphotericin B and oral posaconazole, along with multiple surgeries, the final ocular culture results showed that *F. falciformis* did not grow (Larimore and Libertin, 2025). The above cases suggest that for suspected *F. falciformis* infection, amphotericin B can be used as the cornerstone of antifungal treatment, and triazole antifungal agents can be used as adjunctive therapy. Localized and accessible infection sites, including the skin, sinuses, and eyes, require aggressive surgical debridement or intervention to effectively control *F. falciformis* infection.

*F. verticillioides* is generally associated with plant diseases and is well known as a mycotoxin producer. Before 2020, 11 cases of human *F. verticillioides* infection were documented, involving the nasal cavity, eyes, feet, and bloodstream. From January 2020 to February 2026, only two cases of human infection caused by *F. verticillioides* have been reported. In 2022, *F. verticillioides* was identified via MALDI-TOF MS in the blood of a critically ill COVID-19 patient with normal immune function and multiple skin lesions. This isolate was susceptible to amphotericin B and posaconazole but resistant to voriconazole. Following treatment with sodium deoxycholate amphotericin B and voriconazole, the patient's pulmonary infection was controlled; however, the patient ultimately died of respiratory instability, sepsis, and shock (Barberis et al., 2022). In 2024, a male patient without underlying diseases but with a skin ulcer was found to have *F. verticillioides* in his foot via amplification and sequencing of the TEF1 gene. This isolate was susceptible to amphotericin B, terbinafine, and voriconazole, resistant to micafungin, and showed intermediate susceptibility to itraconazole. Since the patient had normal immune function and the infection remained localized to the skin and nails without deep tissue invasion, itraconazole showed sufficient *in vivo* efficacy. After the patient received antifungal treatment with itraconazole and had a stent implanted in the lower extremity arteries, the ulcer on his skin healed significantly (Figure 2III) (Feng et al., 2024). The above cases suggest that the antifungal susceptibility profiles of *F. verticillioides* may vary significantly among different isolates and even different types of infections. For local infections in individuals with normal immune function, in addition to early and adequate use of highly effective antifungal agents such as liposomal amphotericin B, surgical measures such as debridement, drainage, or vascular reconstruction must also be combined to eliminate the infection site and improve the local microenvironment.

In conclusion, based on the case data collected from January 2020 to February 2026, severe infections caused by *Fusarium spp*. mainly occur in patients with acute myeloid leukemia and in critically ill COVID-19 patients with multiple skin lesions. The global guideline for the diagnosis and management of rare mold infections recommends that for infections caused by *Fusarium spp*., the preferred antifungal agents are voriconazole alone or a combination of voriconazole and amphotericin B (Hoenigl et al., 2021). It is worth noting that in the aforementioned immunocompetent patient, who was treated orally with an antifungal drug with moderate sensitivity as determined by antifungal susceptibility testing, the response was favorable (Feng et al., 2024). From this, it can be inferred that for immunocompetent patients, antifungal susceptibility testing cannot provide absolutely reliable guidance in treating infections caused by *Fusarium spp*..

### 2.3 *Microascus spp*.

*Microascus spp*. are widely distributed in the natural environment, typically found in soil, decaying plant material, indoor environments, and even food (Sandoval-Denis et al., 2016). Generally, they do not pose a threat to immunocompetent individuals. However, as opportunistic pathogens, they can cause severe invasive infections in immunocompromised hosts. With the advancement of molecular diagnostic techniques, clinical reports of rare species among *Microascus spp*., such as *Microascus gracilis* and *Microascus cirrosus*, have been increasing (Ding et al., 2020; Tianprasertkij et al., 2026).

**Figure 3:**
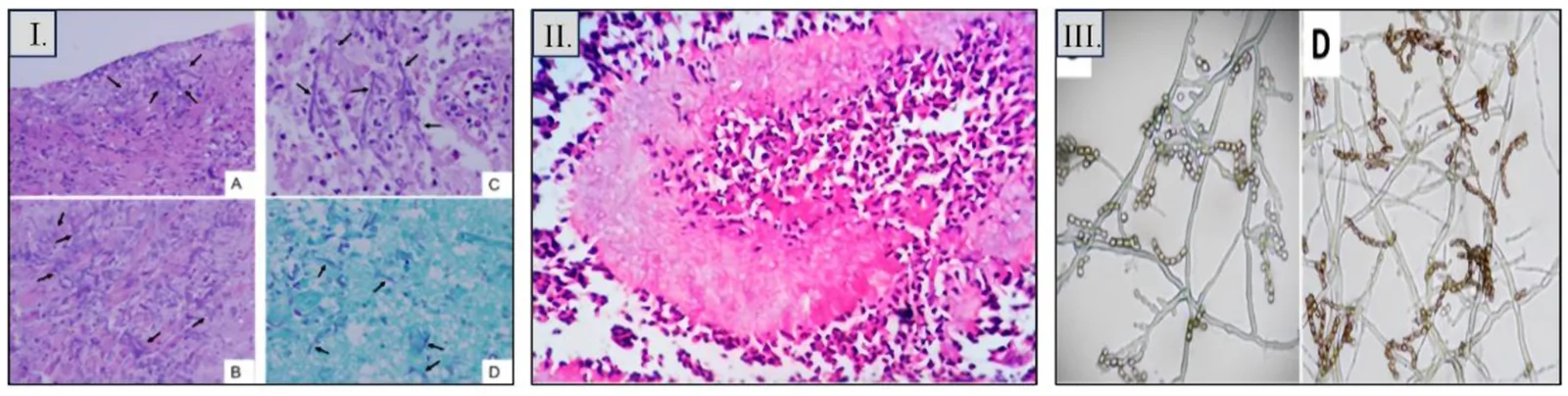
Morphological images of species in the *Microascus spp*. **(I**) Microscopic morphology of *M. gracilis* in pleural effusion specimen (hematoxylin and eosin staining and chromic acid-schiff-diastase special staining) (Ding et al., 2020). **(II)**. Microscopic morphology of *M. gracilis* in foot specimen (Mhmoud et al., 2021). Reprinted from Wang, Y., Liu, Q., Sheng, H., and Yang, S. (2025). Acremonium strictum sphenoid sinusitis in an immunocompetent patient. *Am. J. Case Rep*. 26:e946501. doi: 10.12659/AJCR.946501. **(III)** Microscopic morphology of *M. cirrosus* in abdominal specimen (lactophenol cotton blue staining) (Tianprasertkij et al., 2026). Reprinted from Risum, M., Overgaard, U. M., Rubek, N., Haastrup, E. K., Hare, R. K., Helweg-Larsen, J., et al. (2022). Disseminated fusariosis with cerebral involvement in a patient with acute myeloid leukemia: successful outcome with intrathecal -and systemic antifungal treatment. *J. Infect. Chemother*. 28, 1324–1328. doi: 10.1016/j.jiac.2022.04.017, Feng, Y., Yang, Z., Li, D., Li, J., Li, D., de Hoog, S., et al. (2024). Nails and skin co-infection by Fusarium verticillioides and Proteus vulgaris secondary to arterial occlusion of lower extremity. *Rev. Iberoam. Micol*. 41, 37–42. doi: 10.1016/j.riam.2021.07.003, Mhmoud, N. A., Siddig, E. E., Nyuykonge, B., Bakhiet, S. M., van de Sande, W. W. J., Fahal, A. H., et al. (2021). Mycetoma caused by Microascus gracilis: a novel agent of human eumycetoma in Sudan. *Trans. R. Soc. Trop. Med. Hyg*. 115, 426–430. doi: 10.1093/trstmh/trab010, Samaddar, A., Shrimali, T., Tiwari, S., and Sharma, A. (2023). First report of human infection caused by Curvularia warraberensis, manifesting as invasive sinusitis with intracranial involvement. *J. Mycol. Med*. 33:101337. doi: 10.1016/j.mycmed.2022.101337, with permission from Elsevier.

According to literature searches, limited data are available on the natural habitat and environmental origin of *M. gracilis*. Prior to 2020, *M. gracilis* had never been reported as a cause of disseminated human infection, whereas only two such cases have been reported from January 2020 to February 2026. In 2022, *M. gracilis* was identified from the pleural effusion of a bilateral lung transplant recipient by morphological observation and DNA sequencing of the D1/D2, TUB, and TEF loci. This isolate was susceptible to amphotericin B, isavuconazole, and terbinafine, but resistant to posaconazole and voriconazole. Although the patient received aggressive combination therapy including amphotericin B, isavuconazole, and terbinafine, irreversible intracranial lesions led to the patient's eventual death (Figure 3I) (Ding et al., 2020). In 2021, *M. gracilis* was identified via ITS amplification and sequencing from the left foot lesion of a patient with a history of trauma. Following surgical resection combined with oral itraconazole therapy, the patient achieved complete resolution of mycetoma after 30 months of continuous treatment (Figure 3II) (Mhmoud et al., 2021). The above cases demonstrate a notable discrepancy between the *in vitro* antifungal susceptibility results of *M. gracilis* and its *in vivo* clinical efficacy (Ding et al., 2020; Mhmoud et al., 2021). Given the absence of uniformly effective monotherapy, combined antifungal strategies and surgical debridement serve as the primary treatment strategies for *M. gracilis* infections.

*M. cirrosus* is a rare environmental mold (Figure 3III) (Tianprasertkij et al., 2026). Before 2020, only five cases of human *M. cirrosus* infection had been reported, with isolates recovered from the lungs, skin and other sites. From January 2020 to February 2026, two additional cases have been documented. In 2021, *M. cirrosus* was identified in the lungs of a patient with bronchiectasis via 28S rDNA PCR sequencing. After treatment with voriconazole and amphotericin B, the lung lesions completely disappeared (Liu et al., 2021). In 2026, *M. cirrosus* was identified via ITS sequencing from the abdominal specimen of a patient with end-stage kidney disease. This isolate exhibited extremely high minimum inhibitory concentrations against all tested antifungal agents, indicating a pan-resistant phenotype. First, amphotericin B deoxycholate was administered, followed by voriconazole. The fungal infection was well controlled. The patient continued hemodialysis and remained in stable condition (Tianprasertkij et al., 2026). The above cases suggest that for severe *M. cirrosus* infections, the use of multiple drug combinations for treatment is a common clinical practice, aiming to enhance the therapeutic effect and potentially overcome single-drug resistance. For localized pulmonary infections, in addition to systemic medication, adding aerosol inhalation of antifungal agents can increase the drug concentration at the infection site while reducing systemic toxicity.

In conclusion, *Microascus spp*. are opportunistic pathogenic molds that mainly infect patients with severely compromised immune systems. The prognosis for solid organ transplant recipients is usually poor, while immunocompetent patients with structural lung disease have a relatively better prognosis. The diagnostic challenge of *Microascus spp*. infection lies in the time-consuming culture process and the possibility of confusion with similar isolates. Accurate identification requires molecular techniques (i.e., ITS, TUB, TEF gene sequencing or 28S rDNA PCR). *Microascus spp*. have not been classified as rare molds in the global guideline for the diagnosis and management of rare mold infections, and there are currently no standardized recommended antifungal agents for *Microascus spp*. infections (Hoenigl et al., 2021). Based on the cases summarized above, voriconazole and amphotericin B can be regarded as key agents for treating *Microascus spp*. infections, and they also exert favorable therapeutic effects against multidrug-resistant isolates (Tianprasertkij et al., 2026; Liu et al., 2021). Combining antifungal therapy with surgical debridement can significantly improve clinical outcomes of *Microascus spp*. infections.

### 2.4 *Scedosporium spp*.

*Scedosporium spp*. are molds commonly isolated from soil and contaminated water (Krauth et al., 2021). The most common risk factor for scedosporiosis is severe immunosuppression, and the most frequently observed invasive diseases are those affecting the pulmonary structure (Cortez et al., 2008). In recent years, with advances in molecular diagnostic techniques, clinical cases of rare species among *Scedosporium spp*. have continued to increase, and the epidemiology and clinical features of these species have been gradually better understood.

*Scedosporium dehoogii* is an environmental mold and has been previously isolated from soil (Ramirez-Garcia et al., 2018; Yamazaki et al., 2025). Only two human skin-related infections caused by *S. dehoogii* had been reported before 2020, and two cases caused by this mold have been reported from January 2020 to February 2026. In 2021, *S. dehoogii* was identified via universal broad-range 28S ribosomal polymerase chain reaction (uPCR) in the right knee joint of an immunocompetent male patient with joint damage. The patient recovered after treatment with voriconazole combined with oral terbinafine and supplemented by surgical intervention. This represents the first global case of bone and joint infection caused by *S. dehoogii*, demonstrating that comprehensive strategies including molecular diagnosis, combined antifungal therapy, and surgical intervention can successfully treat infections caused by such rare, drug-resistant isolates (Krauth et al., 2021). In 2025, ITS sequencing identified *S. dehoogii* in the lungs of a patient with acute lymphoblastic leukemia. This isolate displayed high minimum inhibitory concentration (MIC) values against both azoles and echinocandins, confirming its multidrug-resistant phenotype. Following treatment with voriconazole combined with micafungin and subsequent transplant surgery, the patient was successfully cured with no recurrence of the pulmonary fungal infection (Yamazaki et al., 2025). For severe drug-resistant *S. dehoogii* infections, combined antifungal therapy is the key clinical strategy to improve therapeutic outcomes. For trauma patients with an external wound exposure history, even immunocompetent individuals should remain alert to *S. dehoogii* infection.

In conclusion, *Scedosporium spp*. are opportunistic pathogenic molds, and their infections are often underestimated in clinical practice. The main routes of *Scedosporium spp*. infection include traumatic inoculation, spore inhalation, or inhalation after drowning in contaminated water bodies (Hoenigl et al., 2021). Immunocompetent individuals tend to develop localized infections, while immunosuppressed patients are more susceptible to severe infections such as pneumonia, disseminated infections, or infections of the central nervous system. The global guideline for the diagnosis and management of rare mold infections recommends voriconazole as the first-line therapeutic agent for infections caused by *Scedosporium spp*. (Hoenigl et al., 2021). Based on the cases summarized in this article, terbinafine, micafungin, and surgical debridement also play important roles in improving the prognosis of patients infected with *Scedosporium spp*. (Krauth et al., 2021; Yamazaki et al., 2025).

### 2.5 *Paecilomyces spp*.

*Paecilomyces spp*. exist worldwide as saprophytic fungi in soil, where they decompose organic matter, and occasionally serve as insect parasites (Schinabeck and Ghannoum, 2003). Human infections caused by these molds are uncommon and predominate in immunocompromised patients (Marques et al., 2019). Common risk factors include long-term indwelling catheters (i.e., peritoneal dialysis tubes, central venous catheters) and other indwelling medical devices, among which intravenous injection is the most common (Schinabeck and Ghannoum, 2003). People with normal immune function rarely contract *Paecilomyces spp*., but such infections can still occur. In recent years, with the advances in mass spectrometry and molecular diagnostic technologies, rare isolates such as *Paecilomyces variotii* have been increasingly reported.

**Figure 4:**
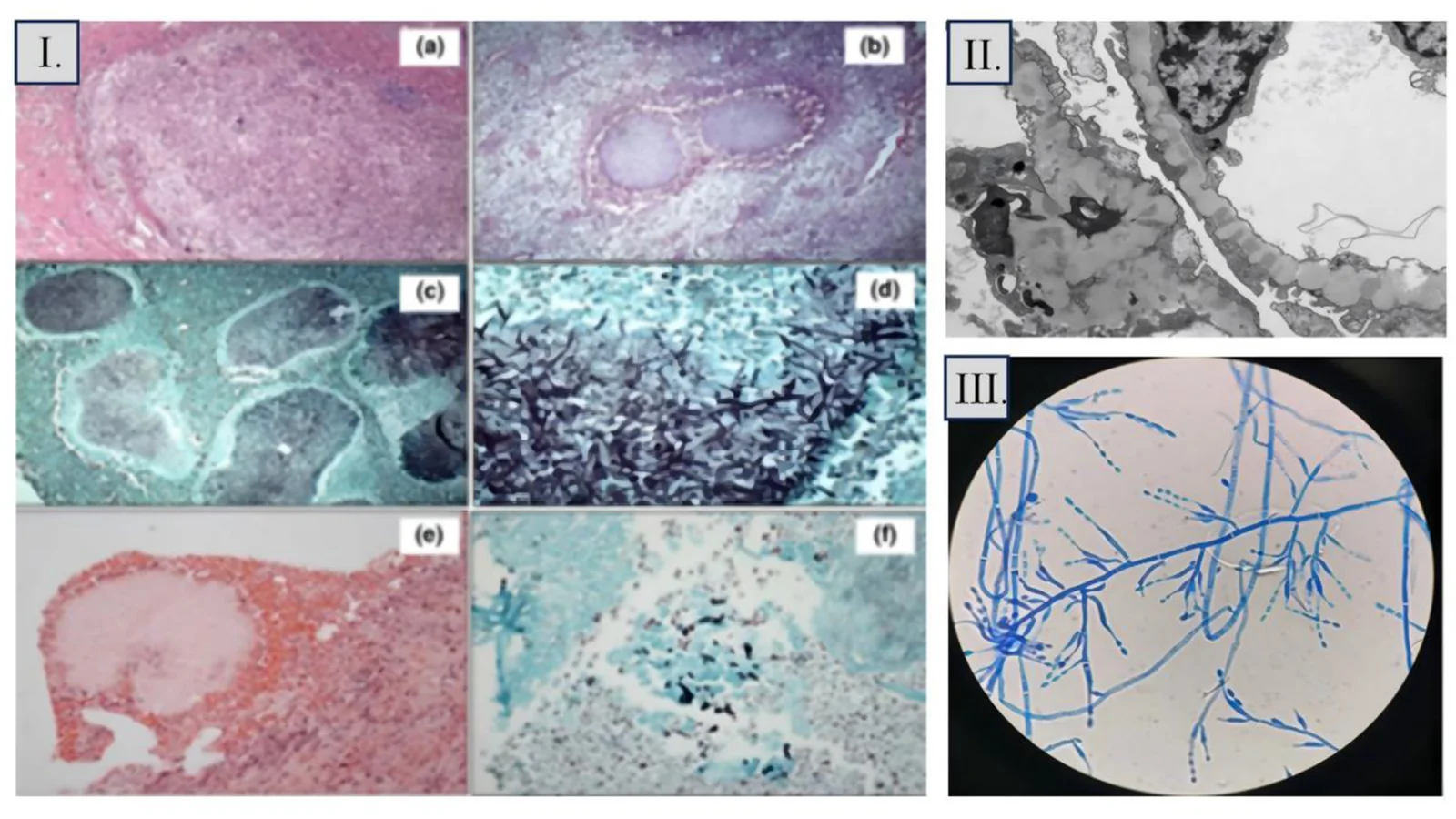
Morphological images of species in the *Paecilomyces spp*. **(I)** Microscopic morphology of *P. variotii* in a hand specimen (hematoxylin and eosin staining and grocott's methenamine silver staining) (Criado et al., 2021). **(II)** Microscopic morphology of *P. variotii* in a lung specimen (Pechacek et al., 2023). **(III)** Microscopic morphology of *P. variotii* in a lung specimen (lactophenol cotton blue staining) (Wang et al., 2024). Reprinted from Shimada, T., Watanabe, A., Akita, K., Bando, K., Takahata, A., Ishikawa, K., et al. (2025). First reported case of disseminated Cunninghamella phaeospora infection with multidrug resistance in acute myeloid leukemia. *J. Infect. Chemother*. 31:102646. doi: 10.1016/j.jiac.2025.102646, with permission from Elsevier.

*P. variotii* is a ubiquitous environmental saprophyte with worldwide distribution. It is commonly found in soil and decomposing organic material (Houbraken et al., 2008). Before 2020, there were 31 reported cases of human infection caused by *P. variotii*, involving the lungs, nose, abdomen, skin, and other body sites. From January 2020 to February 2026, three cases of human infection caused by *P. variotii* have been reported. In 2021, *P. variotii* was isolated from the left hand of a patient with type 2 diabetes via tissue culture. The patient initially received itraconazole monotherapy; following drug discontinuation, liposomal amphotericin B was given alongside surgical intervention, and subsequent combined therapy with itraconazole and terbinafine eventually improved the patient's skin lesions with gradual resolution of local discharges. Following a new partial lesion excision 1 year later, no recurrence was observed during a 3-year follow-up (Figure 4I) (Criado et al., 2021). In 2023, *P. variotii* was detected in the lungs of a patient with systemic lupus erythematosus using MALDI-TOF MS. This isolate was highly susceptible to amphotericin B, itraconazole, and posaconazole. After treatment with posaconazole first and then micafungin, the inflammatory condition in the patient's lungs improved significantly (Figure 4II) (Pechacek et al., 2023). In 2024, *P. variotii* was identified in the lungs of a patient with type 2 diabetes by ITS sequencing; the isolate was resistant to voriconazole but susceptible to amphotericin B, and 4 weeks of liposomal amphotericin B therapy led to complete resolution of pneumonia and subsequent hospital discharge (Figure 4III) (Wang et al., 2024).

In conclusion, infections caused by *Paecilomyces spp*. primarily occur in immunocompromised individuals, including patients with diabetes, systemic lupus erythematosus, and long-term users of immunosuppressive agents. Infections caused by *Paecilomyces spp*. are rare and difficult to manage. Differential diagnosis requires distinguishing them from other filamentous fungi such as *Penicillium spp.*, while techniques including MALDI-TOF MS and ITS sequencing allow accurate identification of these molds. The global guideline for the diagnosis and management of rare mold infections recommends liposomal amphotericin B as the first-line therapeutic agent for infections caused by *Paecilomyces spp*. (Hoenigl et al., 2021). Furthermore, from the above cases, it can be seen that posaconazole, due to its excellent *in vitro* activity, can be regarded as an effective oral drug option for treating infections caused by *Paecilomyces spp*. (Pechacek et al., 2023).

### 2.6 *Cladophialophora spp*.

*Cladophialophora spp*. are widely distributed in soil, decaying plants, and organic matter, especially in tropical and subtropical regions (Jain et al., 2018). Accurate identification of *Cladophialophora spp*., particularly cryptic and closely related species, relies heavily on molecular techniques, with ITS sequencing being the most widely used and reliable approach.

**Figure 5:**
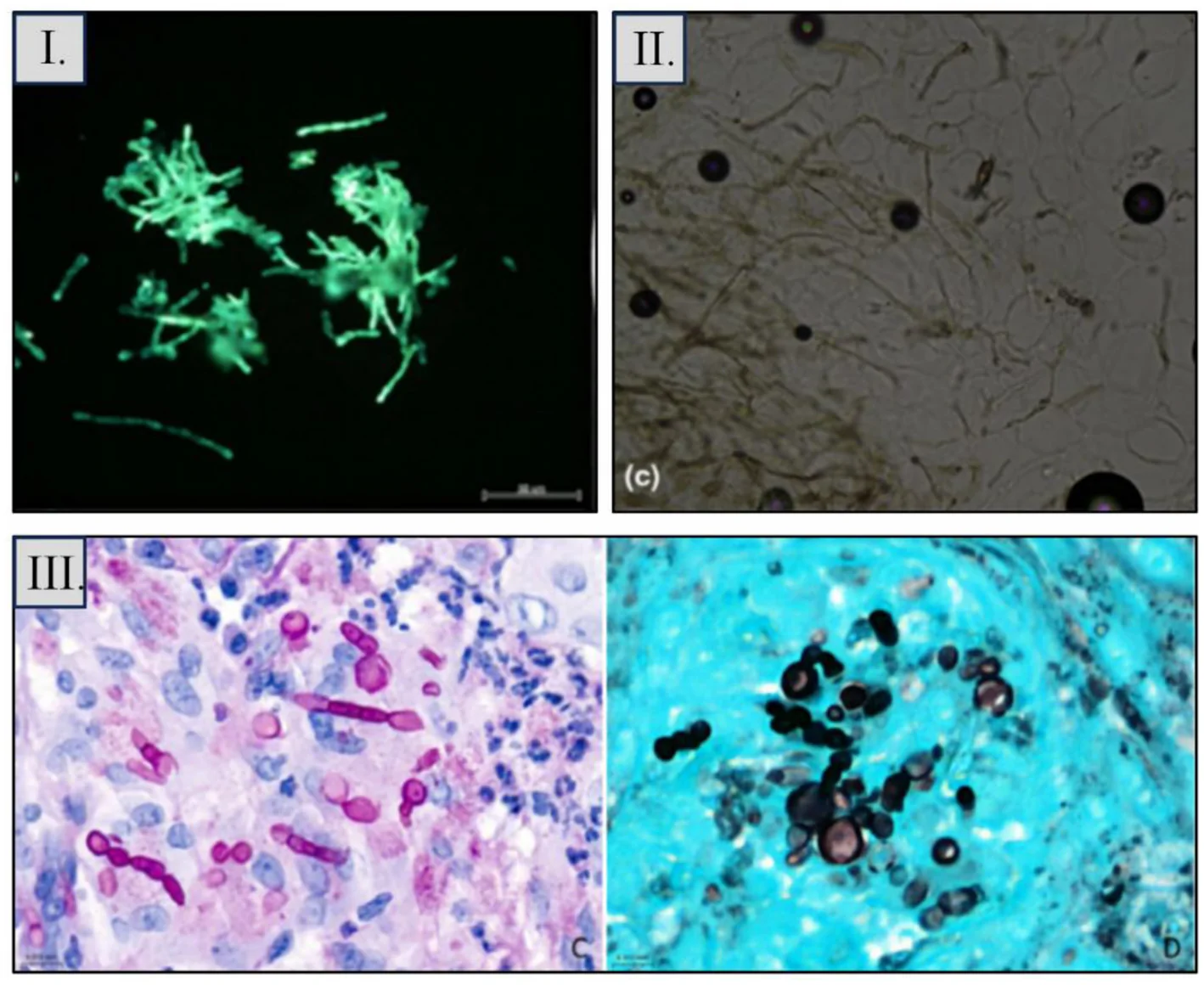
Morphological images of species in the *Cladophialophora spp*. **(I)** Microscopic morphology of *C. boppii* in a cornea specimen (calcofluor staining) (Fukuoka et al., 2024). **(II)** Microscopic morphology of *C. boppii* in a fingernail specimen (Fukuyama and Yaguchi, 2024). **(III)** Microscopic morphology of *C. boppii* in a skin specimen (periodic acid-schiff diastase staining and grocott's methenamine silver staining) (Cavens et al., 2025). Reprinted from Semis, M., Dadwal, S. S., Tegtmeier, B. R., Wilczynski, S. P., Ito, J. I., Kalkum, M., et al. (2021). First case of invasive Stachybotrys sinusitis. *Clin. Infect. Dis*. 72, 1386–1391. doi: 10.1093/cid/ciaa231, with permission from Elsevier.

According to the literature search, no specific information was provided regarding the natural habitat or environmental origin of *Cladophialophora boppii*.. It is reported that *C. boppii.*, a dematiaceous fungus that rarely causes human infectious diseases, was first isolated from a Brazilian woman suffering from chromomycosis by C. Bopp in 1983 (Bopp, 1983). Before 2020, only three cases of human *C. boppii* infection were reported, primarily affecting the nails, skin and feet. From January 2020 to February 2026, three cases of human infection caused by *C. boppii* have been reported. In 2024, *C. boppii* was identified via DNA sequencing in the cornea of an immunocompetent male; the isolate exhibited susceptibility to micafungin and 5-flucytosine, yet resistance to amphotericin B, fluconazole, itraconazole, and voriconazole. The patient received topical 0.1% miconazole eye drops and natamycin ointment, combined with oral itraconazole, which was subsequently discontinued because of diarrhea. Three weeks later, the patient developed corneal perforation and refused surgery, maintaining topical therapy for 8 months. After treatment discontinuation, mold inflammation subsided, yet permanent corneal scarring and persistent perforation remained, resulting in a severely impaired visual prognosis (Figure 5I) (Fukuoka et al., 2024). In 2024, a 61-year-old immunocompetent male presented with a 6-month history of gradually expanding brown streaks on the right great toenail. Through ITS sequencing, the pathogen *C. boppii* was detected in the patient's nails. After 8 weeks of 5% luliconazole treatment, the patient achieved complete resolution of longitudinal melanonychia (Figure 5II) (Fukuyama and Yaguchi, 2024). In 2025, *C. boppii* was identified by ITS1 and ITS4 sequencing in a skin lesion from a patient with stage IV breast cancer. This isolate was susceptible to most azoles, echinocandins and terbinafine, yet resistant to fluconazole. After the complete removal of the skin lesion and oral administration of itraconazole, no new skin lesions were observed during the 4-month follow-up period (Figure 5III) (Cavens et al., 2025).

In conclusion, infection with *Cladophialophora spp*. has been observed in both immunocompromised individuals and those with normal immune function. Clinically, these molds commonly cause a wide spectrum of human infections, ranging from mild skin lesions to fatal encephalitis (Badali et al., 2008). For patients who have been using immunosuppressants for a long time, if they develop atypical skin patches, corneal black spots or nail plate pigment streaks, they should be highly vigilant for *C. boppii* infection. According to the global guideline for the diagnosis and management of rare mold infections, *Cladophialophora spp*. have not been classified as rare molds, and no antifungal agents are recommended for infections caused by them (Hoenigl et al., 2021). Based on the cases summarized above, when facing a suspected *C. boppii* infection, the importance of thoroughly eliminating or locally suppressing the pathogen through physical means such as surgery or topical drug administration is no less than that of relying solely on systemic antifungal chemotherapy (Fukuoka et al., 2024). Furthermore, based on antifungal susceptibility testing results, itraconazole is effective against infections caused by *C. boppii* (Cavens et al., 2025).

### 2.7 *Talaromyces spp*.

*Talaromyces spp*. are a group of genetically diverse molds commonly found in the natural environment, primarily inhabiting soil (Todokoro et al., 2023). *Talaromyces marneffei* is a highly pathogenic species among *Talaromyces spp*., capable of causing disseminated, fatal infections. Other species within *Talaromyces spp*., though relatively rare, can still cause severe invasive infections in severely immunocompromised hosts. In recent years, infections caused by non*-marneffei Talaromyce*s *spp*. have been increasingly reported; these species typically induce invasive infections but are far less common than *T. marneffei*.

**Figure 6:**
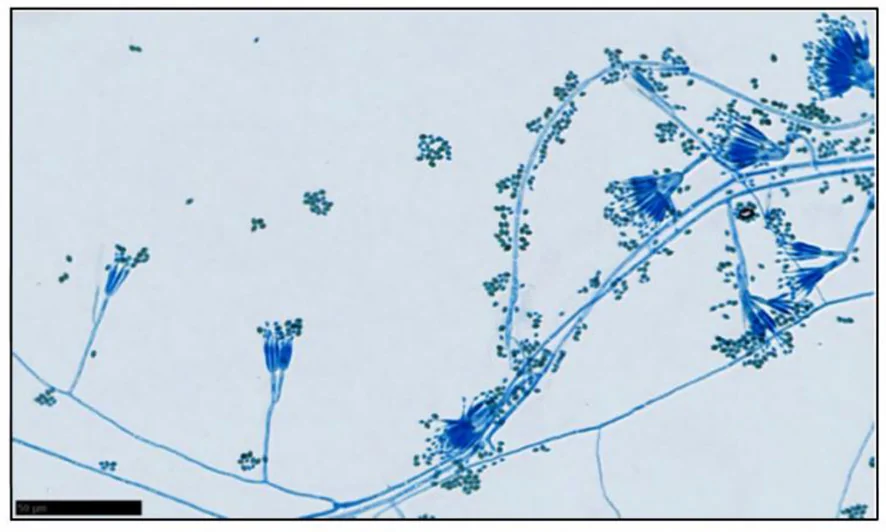
Morphological images of species in the *Talaromyces spp*. Microscopic morphology of *T. coalescens* in an eye specimen (lactophenol cotton blue staining) (Todokoro et al., 2023).

According to the literature search, no specific information was provided regarding the natural habitat or environmental origin of *Talaromyces coalescens*. Prior to 2020, no human infections due to *T. coalescens* had been reported; only a single case has been documented thereafter. In 2023, *T. coalescens* was detected by ITS sequencing in a 77-year-old male with multiple underlying ocular disorders. The isolate showed susceptibility to natamycin and resistance to voriconazole and fluconazole. After 2 months of treatment with 5% natamycin eye drops, the corneal ulcer improved clinically (Figure 6) (Todokoro et al., 2023). This is the first globally reported case of human infection caused by *T. coalescens*, with no severe infections having occurred to date.

In conclusion, patients with corneal diseases, long-term users of topical glucocorticoids or immunosuppressants, contact lens wearers, and individuals with a history of ocular trauma or surgery should remain alert to *T. coalescens* infection. The global guideline for the diagnosis and management of rare mold infections recommends liposomal amphotericin B as the first-line therapy for *non-marneffei Talaromyces spp*. infections (Hoenigl et al., 2021). Based on the analysis of the above cases, natamycin may be an effective option for treating *T. coalescens* keratitis (Todokoro et al., 2023).

### 2.8 *Sarocladium spp*.

*Sarocladium spp*. are filamentous fungi that are usually associated with plant diseases and rarely cause human diseases (Gaspar et al., 2025; Pérez-Cantero and Guarro, 2020). With advances in clinical laboratory testing, rare strains within *Sarocladium spp*., including *Sarocladium strictum*, have been increasingly identified as human pathogens (Cui et al., 2021). Immunocompromised patients have a risk of developing this infections, which usually manifests as skin and subcutaneous lesions, and may eventually spread to internal organs (Gaspar et al., 2025). The identification of *Sarocladium spp*. is challenging and can often lead to diagnostic delays. Molecular methods are indispensable for differentiating *Sarocladium spp*. from other pathogenic fungi, such as *Fusarium spp* (Cui et al., 2021).

**Figure 7:**
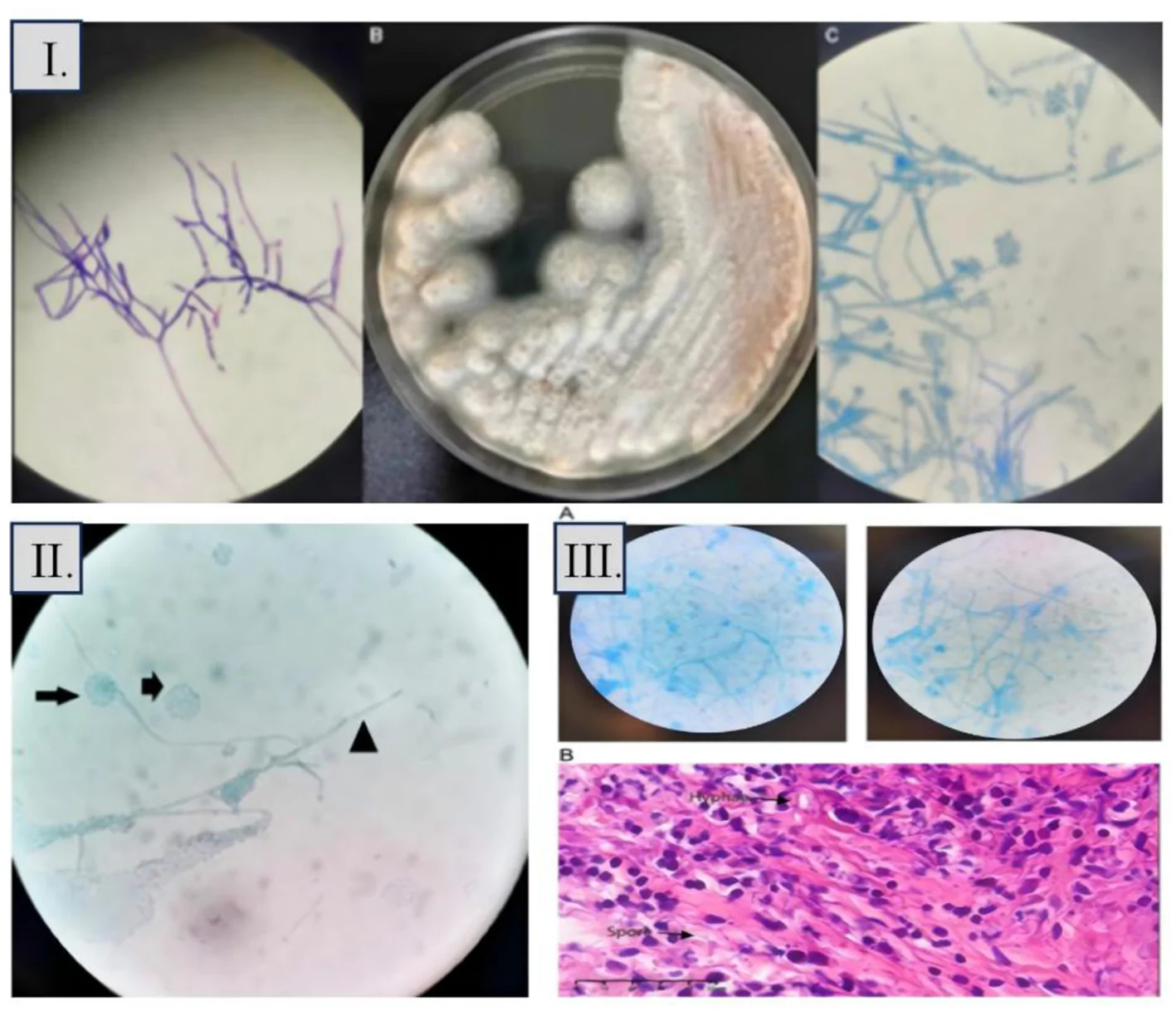
Morphological images of species in the *Sarocladium spp*. **(I)** The microscopic morphology and colony culture of *S. strictum* in a cerebrospinal fluid specimen (gram stain and lactophenol cotton blue staining) (Cui et al., 2021). **(II)** Microscopic morphology of *S. strictum* in a nasal specimen (lactophenol cotton blue staining) (Wang et al., 2025). **(III)** Microscopic morphology of *S. strictum* in a cerebrospinal fluid specimen (lactophenol cotton blue staining) (Huang et al., 2026).

*S. strictum*, reclassified from *Acremonium spp*. by Summerbell et al. in 2011 based on rDNA sequence analysis, is a ubiquitous saprophytic fungus that commonly infects plants and insects (Perdomo et al., 2011). *S. strictum* was initially isolated from common wheat (Giraldo et al., 2015). Prior to 2020, there were no reports of human infection caused by *S. strictum*, and only three such cases have been reported worldwide from January 2020 to February 2026. In 2021, mNGS and ITS sequencing identified *S. strictum* in the cerebrospinal fluid of a patient with spontaneous rhinorrhea. At admission, the patient was treated with combined amphotericin B and 5-fluorocytosine; 5-fluorocytosine was later discontinued with voriconazole added, followed by amphotericin B monotherapy. Though the clinical condition did not deteriorate, the patient continued to have persistent headaches and blurred vision (Figure 7I) (Cui et al., 2021). When identifying *S. strictum*, mNGS enabled rapid, unbiased pathogen screening and detection, while ITS sequencing accurately identified the species of cultured isolates. The combination of these two molecular identification techniques enabled the precise identification of *S. strictum*. In 2025, *S. strictum* was detected in the nasal cavity of an immunocompetent female patient via 28S rRNA amplification and sequencing. She received oral voriconazole for 4 weeks, and a 2-year follow-up confirmed complete resolution of sphenoid sinusitis without recurrence (Figure 7II) (Wang et al., 2025). In 2026, in the cerebrospinal fluid of a patient with sarcoidosis, *S. strictum* was identified through mNGS analysis. After treatment with voriconazole and liposomal amphotericin B, the mold infection in the patient was controlled at the drug level, but the patient ultimately died of cerebral ischemia (Figure 7III) (Huang et al., 2026).

In conclusion, the identification of *Sarocladium spp*. is challenging and often leads to delays in timely diagnosis. Molecular methods are essential for differentiating *Sarocladium spp*. from other pathogenic fungi, such as *Fusarium spp*. (Pérez-Cantero and Guarro, 2020). When dealing with these rare and dangerous isolate of *S. strictum*, the diagnosis should primarily rely on molecular techniques such as mNGS or targeted gene sequencing to overcome the limitations of traditional methods. In the global guideline for the diagnosis and management of rare mold infections, *Sarocladium spp*. have not been classified as rare molds, and no antifungal agents are recommended for this type of infection (Hoenigl et al., 2021). Based on the cases summarized above, amphotericin B combined with voriconazole is the core treatment strategy currently supported by clinical evidence for infections caused by *S. strictum* (Huang et al., 2026).

### 2.9 *Nannizziopsis spp*.

The natural habitat and distribution of *Nannizziopsis spp*. have not yet been determined. However, several species of this genus can infect the skin of various lizards, including green iguanas, inland and coastal bearded dragons, and leopard geckos, thereby inducing local and systemic infections (Abarca et al., 2010; Schneider et al., 2018; Sigler et al., 2013; Stchigel et al., 2013; Lai et al., 2024). In recent years, advances in clinical laboratory techniques have led to the identification of rare species within *Nannizziopsis spp*., which have been reported to cause human infections.

**Figure 8:**
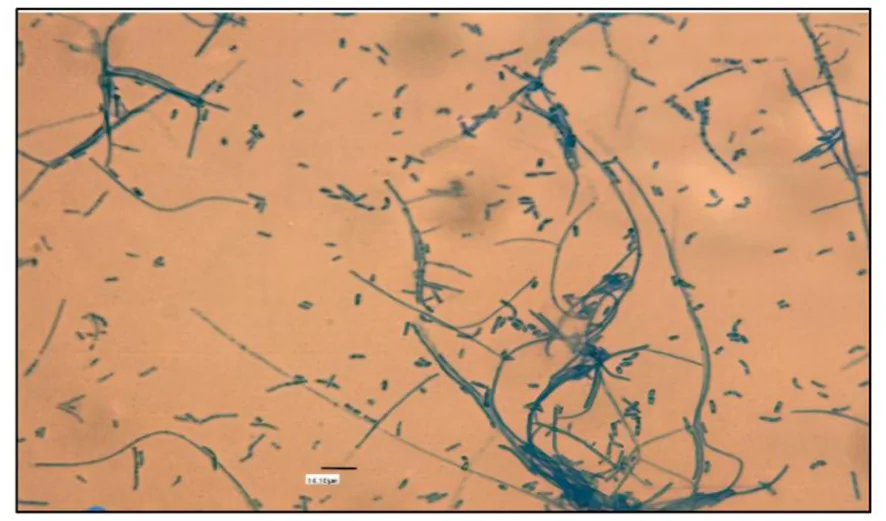
Morphological images of species in the *Nannizziopsis spp*. Microscopic morphology of *N. obscura* in a breast specimen (lactophenol cotton blue staining) (Jiménez-García et al., 2023).

The ecology of *Nannizziopsis obscura* remains unclear. Prior to 2020, there were no reports of human infection caused by *N. obscura*, and only three such cases have been reported globally from January 2020 to February 2026. In 2022, *N. obscura* was isolated from the joint tissue of a kidney transplant patient via ITS and MALDI-TOF MS analysis, and this strain exhibited *in vitro* susceptibility to all commonly tested antifungal agents. After 1 year of treatment with posaconazole, the patient did not experience any recurrence of sepsis, and the function of the right lower limb partially recovered (Mascitti et al., 2022). ITS sequencing is a type of genotyping, while MALDI-TOF MS is a form of phenotyping. These two techniques operate on entirely different principles, and their combined use enables result cross-verification and overcomes the inherent limitations of a single method for identifying *N. obscura*. In 2023, *N. obscura* was isolated from the right breast of a patient with a prior kidney transplant via ITS sequencing, and the isolate showed *in vitro* susceptibility to all commonly tested antifungal agents. Surgical drainage combined with short-term isavuconazole treatment successfully controlled the breast abscess caused by *N. obscura*. No recurrence was observed during the six-month follow-up (Figure 8) (Jiménez-García et al., 2023). In 2024, genetic sequencing was conducted on specimens collected from the leg of a patient with a prior kidney transplantation, and *N. obscura* was detected. The patient received surgical drainage combined with short-term isavuconazole treatment; however, the clinical condition continued to deteriorate. Consequently, the patient was transferred to palliative care (Soyele et al., 2024).

In conclusion, in the above cases, infections caused by *N. obscura* predominantly occurred in heavily immunosuppressed patients, especially those with solid organ transplants, most commonly kidney transplantation. In clinical practice, when immunosuppressed patients with a history of travel to Africa develop soft tissue or bone and joint infections, *N. obscura* infection should be highly suspected. For the identification of *N. obscura*, ITS sequencing is the gold standard for diagnosis, and MALDI-TOF MS can also serve as an auxiliary method when the database is well-established. The global guideline for the diagnosis and management of rare mold infections has not yet classified *Nannizziopsis spp*. as rare molds, nor has it recommended antifungal agents for such infections (Hoenigl et al., 2021). According to the antifungal susceptibility testing in these case reports, *N. obscura* exhibits high susceptibility to commonly used azole antifungal agents, including voriconazole, posaconazole, itraconazole, and isavuconazole. Combining antifungal therapy with early and aggressive surgical debridement or drainage enables effective treatment of *N. obscura* infections (Mascitti et al., 2022; Jiménez-García et al., 2023).

### 2.10 *Chondrostereum spp*.

*Chondrostereum spp*. were collected from the soft coral *Sarcophyton tortuosum* in the South China Sea (Li et al., 2012). In recent years, several reports have indicated that certain rare *Chondrostereum species* can cause human infections.

**Figure 9:**
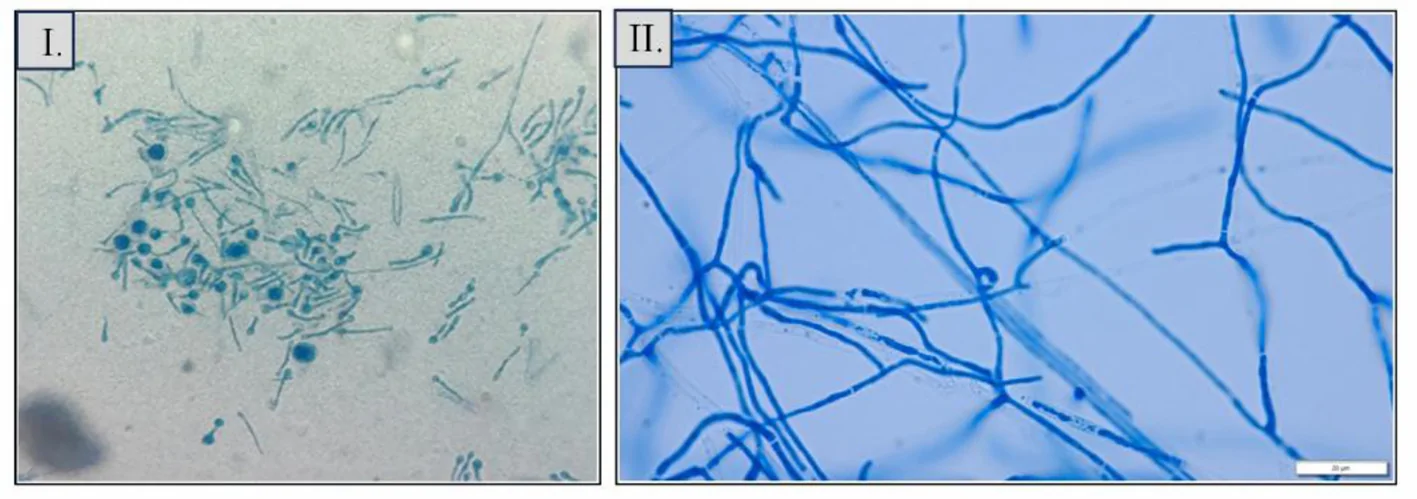
Morphological images of species in the *Chondrostereum spp*. **(I)** Microscopic morphology of *C. purpureum* in a paratracheal abscess specimen (lactophenol cotton blue staining) (Dutta and Ray, 2023). **(II)** Microscopic morphology of *Chondrostereum purpureum* in a shoulder specimen (lactophenol cotton blue staining) (Kijacharoenchai et al., 2026).

*Chondrostereum purpureum* is a plant fungus that causes silver leaf disease, particularly in members of the genus Rosa (Figure 9I) (Dutta and Ray, 2023). Prior to 2020, no human infections caused by *C. purpureum* had been documented; however, two cases have been reported from January 2020 to february 2026. In 2023, during an examination of the trachea of a male with normal immune function, *C. purpureum* was identified through DNA sequencing. After oral voriconazole therapy and surgical drainage, the patient was followed up for 2 years and there was no recurrence of the paratracheal abscess (Dutta and Ray, 2023). In 2026, *C. purpureum* was detected in the right shoulder of a female patient with a previous kidney transplant via ITS and D1/D2 sequencing. After oral posaconazole administration and surgical drainage, the patient was followed up for 1 year, with no signs of subcutaneous abscess recurrence (Figure 9II) (Kijacharoenchai et al., 2026). These are the first two documented human cases of infection caused by *C. purpureum*. Since the antifungal susceptibility profile of this mold has not been previously investigated or reported in the literature, treatment decisions were based on limited clinical outcomes and the general antifungal susceptibility patterns of related pathogens (Chowdhary et al., 2014).

In conclusion, within the global guideline for the diagnosis and management of rare mold infections, *Chondrostereum spp*. have not been classified as rare molds, and evidence-based treatment recommendations are not yet available (Hoenigl et al., 2021). For rare infections caused by *C. purpureum*, clinical outcomes from the above cases indicate that broad-spectrum azoles (voriconazole and posaconazole), combined with surgical debridement and drainage, constitute the core treatment strategy for such infections (Dutta and Ray, 2023; Kijacharoenchai et al., 2026).

### 2.11 Others

In addition to the aforementioned molds, a literature search identified eight rare molds that had never been reported to cause human case of infection prior to 2020. Over the past 6 years, only a single case has been recorded for each mold, with each representing the first documented human infection. Before being first identified as human pathogens, these molds were primarily regarded as plant pathogens, environmental saprophytes, or animal pathogens.

**Figure 10:**
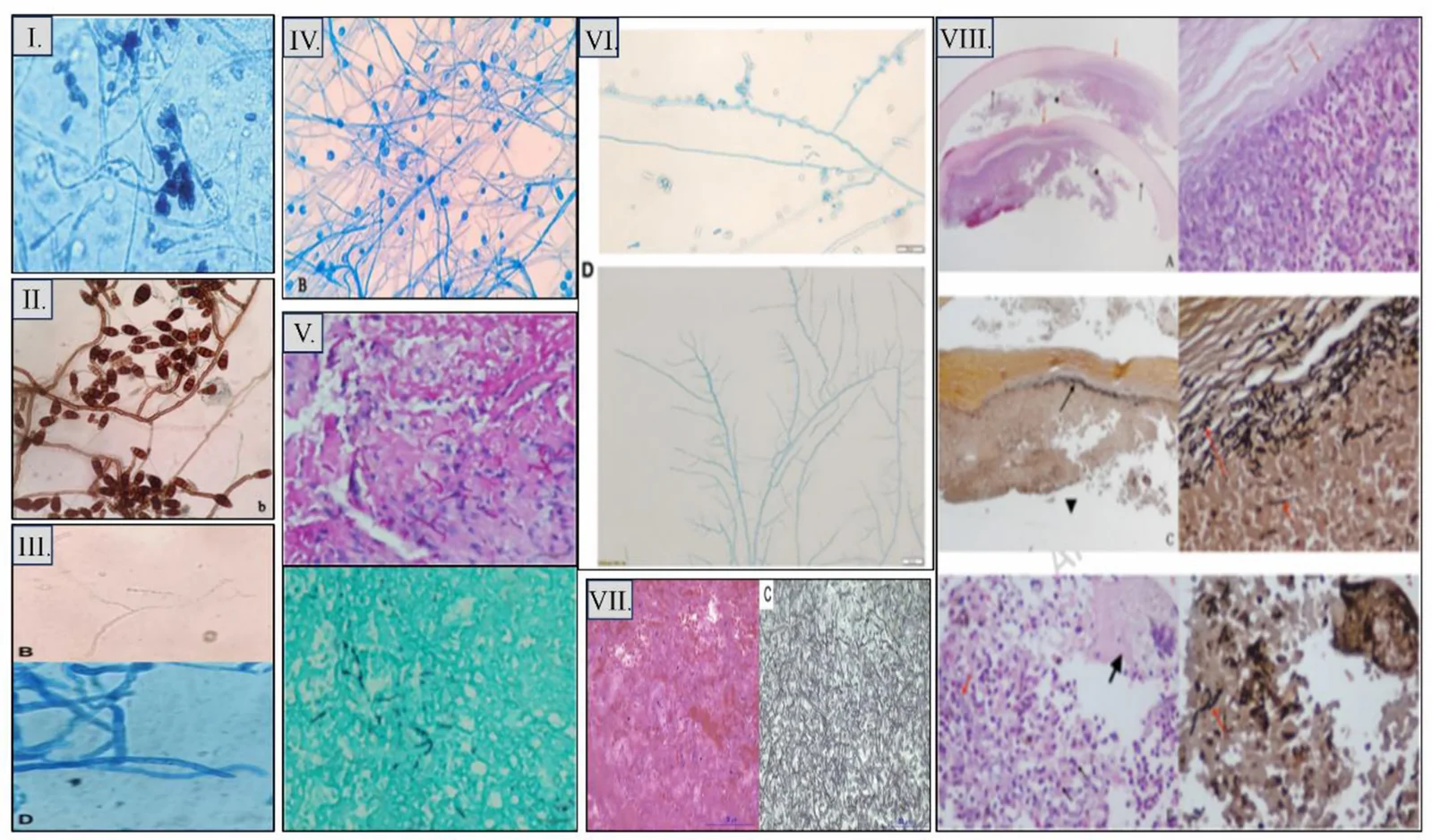
Other rare molds morphological images. **(I)** Microscopic morphology of *S. chlorohalonata* in a paranasal sinus specimen (lactophenol cotton blue staining) (Semis et al., 2021). **(II)** Microscopic morphology of *C. warraberensis* in a paranasal sinus specimen (lactophenol cotton blue staining) (Samaddar et al., 2023a). (**III)** Microscopic morphology of *C. samala* in a cornea specimen (lactophenol cotton blue staining) (Gupta et al., 2022). (**IV)** Microscopic morphology of *M. setulosa* in a foot tissue specimen (lactophenol cotton blue staining) (Samaddar et al., 2023a). **V**.Microscopic morphology of *F. micropora* in a leg specimen. (H&E staining and GMS staining) (Jarrin Jara et al., 2025). (**VI)** Microscopic morphology of *T. elegans* and *P. incarnata* in a sputum specimen (lactophenol cotton blue staining) (Matsuo et al., 2025)**. (VII)** Microscopic morphology of *C. phaeospora* in a lung specimen (hematoxylin and eosin staining) (Shimada et al., 2025). **(VIII)** Microscopic morphology of *D. aspalathi* in a cornea specimen [(HE staining: × 20, × 400; GMS staining: × 40, × 400)]. (Huang et al., 2025).

In 2021, *Stachybotrys chlorohalonata*, which exhibited susceptibility to liposomal amphotericin B and micafungin but resistance to voriconazole and posaconazole, was isolated from the paranasal sinus of a patient with acute lymphoblastic leukemia. After undergoing sinus surgery and combined antifungal therapy with amphotericin B lipid complex and micafungin, the patient received a second biopsy, which yielded a negative result. However, the patient later developed invasive sinusitis caused by *Aspergillus calidoustus* and ultimately died (Figure 10I) (Semis et al., 2021). Patients with severely compromised immune systems may develop successive infections by different molds with distinct resistance profiles. After the control of *S. chlorohalonata* infection, close monitoring for secondary infections remains essential. In 2022, *Curvularia warraberensis* was identified via ITS sequencing in the paranasal sinus of an immunocompetent patient with a history of tuberculosis; this isolate of which was found to be susceptible to posaconazole, itraconazole, and amphotericin B, moderately susceptible to voriconazole, and resistant to fluconazole. After undergoing endoscopic sinus surgery and bilateral sphenoethmoidectomy, the patient received combined antifungal treatment with liposomal amphotericin B and itraconazole. However, the patient eventually died of respiratory failure. The death was attributed to extensive irreversible intracranial damage resulting from delayed presentation and diagnosis, rather than antifungal treatment failure (Figure 10II) (Samaddar et al., 2023a,b). Patients with chronic sinusitis accompanied by intracranial symptoms associated with *C. warraberensis* should receive timely histopathological examination and molecular identification, even with normal immune function, to prevent delayed diagnosis and treatment. In 2022, *Cladorrhinum samala* was detected in the corneal specimen from an immunocompetent male with ocular trauma through PCR amplification of the ribosomal DNA internal transcribed spacer (ITS) region using the ITS-1 and ITS-4 primer pairs, followed by Sanger sequencing.The patient received combined therapy with natamycin eye drops, voriconazole eye drops and mydriatic agents, which resulted in complete resolution of hypopyon within 1 week. Although corneal scarring caused visual loss, the eyeball was retained, and no disease recurrence was observed over a 1-year follow-up period (Figure 10III) (Gupta et al., 2022). In the absence of antifungal susceptibility testing data, combined topical use of natamycin and voriconazole can be regarded as a good treatment option for keratitis caused by *C. samala*. In 2023, a male with normal immune function visited the hospital due to multiple painful swollen lesions on his left foot. Through DNA sequencing, researchers discovered *Megasporoporia setulosa* in the skin sample from his foot. *M. setulosa* was susceptible to voriconazole, posaconazole, itraconazole, isavuconazole, and amphotericin B but was resistant to fluconazole. Following surgical excision of the lesion combined with antifungal therapy involving amphotericin B deoxycholate and itraconazole, the lesion completely resolved, and no recurrence was observed during a 3-month follow-up (Figure 10IV) (Samaddar et al., 2023a). Although *M. setulosa* shows susceptibility to voriconazole and itraconazole, fluconazole and echinocandins are not recommended. Surgical debridement remains the mainstay of therapy for subcutaneous soft tissue infections. In 2025, *Fomitiporella micropora* was detected via ITS and 28S rRNA sequencing in the right lower leg of a heart transplant recipient. The patient underwent complete surgical resection of the mass, followed by oral posaconazole treatment. Follow-up after drug discontinuation confirmed complete lesion healing (Figure 10V) (Jarrin Jara et al., 2025). For *F. micropora*, a rare mold newly reported to cause human infection, the use of two conserved gene regions for molecular identification can improve the accuracy and reliability of diagnostic results. For infections caused by this pathogen, surgical resection may be essential for complete cure, while antifungal agents such as posaconazole serve as an important adjunctive therapy. In 2025, *Trametes elegans and Peniophora incarnata* were identified in the sputum of an advanced acquired immunodeficiency syndrome (AIDS) patient through ITS and 28S rDNA sequencing. After treatment with liposomal amphotericin B, the patient's condition deteriorated; however, following a switch to voriconazole, respiratory symptoms resolved, pulmonary nodules shrank, and the patient ultimately improved (Figure 10VI) (Matsuo et al., 2025). *T. elegans* and *P. incarnata* had not been previously recognized as human pathogens, and standard clinical microbiology laboratories lack the capacity to detect or classify such rare molds; however, the two molds were accurately identified using the above two molecular approaches. Representing the first documented human infection caused by these isolates, this finding also indicates that voriconazole may offer superior therapeutic efficacy compared with amphotericin B for their associated pulmonary infections. In 2025, *Diaporthe aspalathi* was detected via mNGS in the cornea of an immunocompetent woman with a prior left ocular injury. Topical voriconazole eye drops combined with oral itraconazole yielded limited therapeutic effects. Following penetrating keratoplasty, the inferior cornea developed scarring, vascularization and thinning, with the patient's vision reduced to only light perception. Fungal cultures for *D. aspalathi* were negative after 2 weeks of observation. Although corneal transplantation was recommended to improve visual acuity, the patient declined the procedure (Figure 10VIII) (Huang et al., 2025). *D. aspalathi* infection usually requires surgical debridement combined with multiple local and systemic antifungal treatments. Simple drug therapy often fails to completely eradicate the deep colonized hyphae of *D. aspalathi*. In 2025, a patient with acute myeloid leukemia and type 2 diabetes was hospitalized for pancytopenia. Suspected of mucormycosis, the patient received isavuconazole; after clinical deterioration, liposomal amphotericin B was administered. Nevertheless, the patient died on the 19th hospital day due to respiratory and multiple organ failure. Autopsy specimens from the lung tested positive for *Cunninghamella phaeospora* via ITS and D1/D2 sequencing, and the strain showed broad resistance to all mainstream antifungal agents currently available (Figure 10VII) (Shimada et al., 2025). For the multidrug-resistant isolate of *C. phaeospora*, even when standard doses of liposomal amphotericin B and the novel azole isavuconazole are administered, drug therapy may be completely ineffective in the absence of effective immune restoration.

In conclusion, these newly emerging human-pathogenic rare molds have been identified not only in severely immunocompromised patients but also in immunocompetent individuals. Rare mold infections can occur in immunocompetent hosts, suggesting that high-risk occupational exposures, such as farming, gardening, and plant-related trauma, may serve as independent and critical risk factors. The eight rare molds mentioned above are not featured in the global guideline for the diagnosis and management of rare mold infections, and there are currently no recommended first-line antifungal agents for them. In the aforementioned cases, the common feature of the successful ones was that the diagnosis was made relatively promptly and combined treatment with surgery and targeted antifungal agents was implemented (Hoenigl et al., 2021). It can be inferred that for the above-mentioned rare mold infections, surgical debridement is the cornerstone, while drugs serve as adjunctive therapy (Samaddar et al., 2023a; Jarrin Jara et al., 2025; Huang et al., 2025). In the face of these rare mold infections, it is not advisable to rely on empirical treatment for *Aspergillus* or *Candida*. Before treatment, isolates must be obtained and antifungal susceptibility testing must be conducted to guide precise treatment. This group of molds, traditionally regarded as non-pathogenic environmental molds, can cross species barriers under specific conditions—such as immunosuppression or mucosal/skin barrier disruption—and emerge as rare human pathogens. Collectively, they represent the expanding spectrum of rare and emerging opportunistic pathogens in clinical mycology.

**Figure 11:**
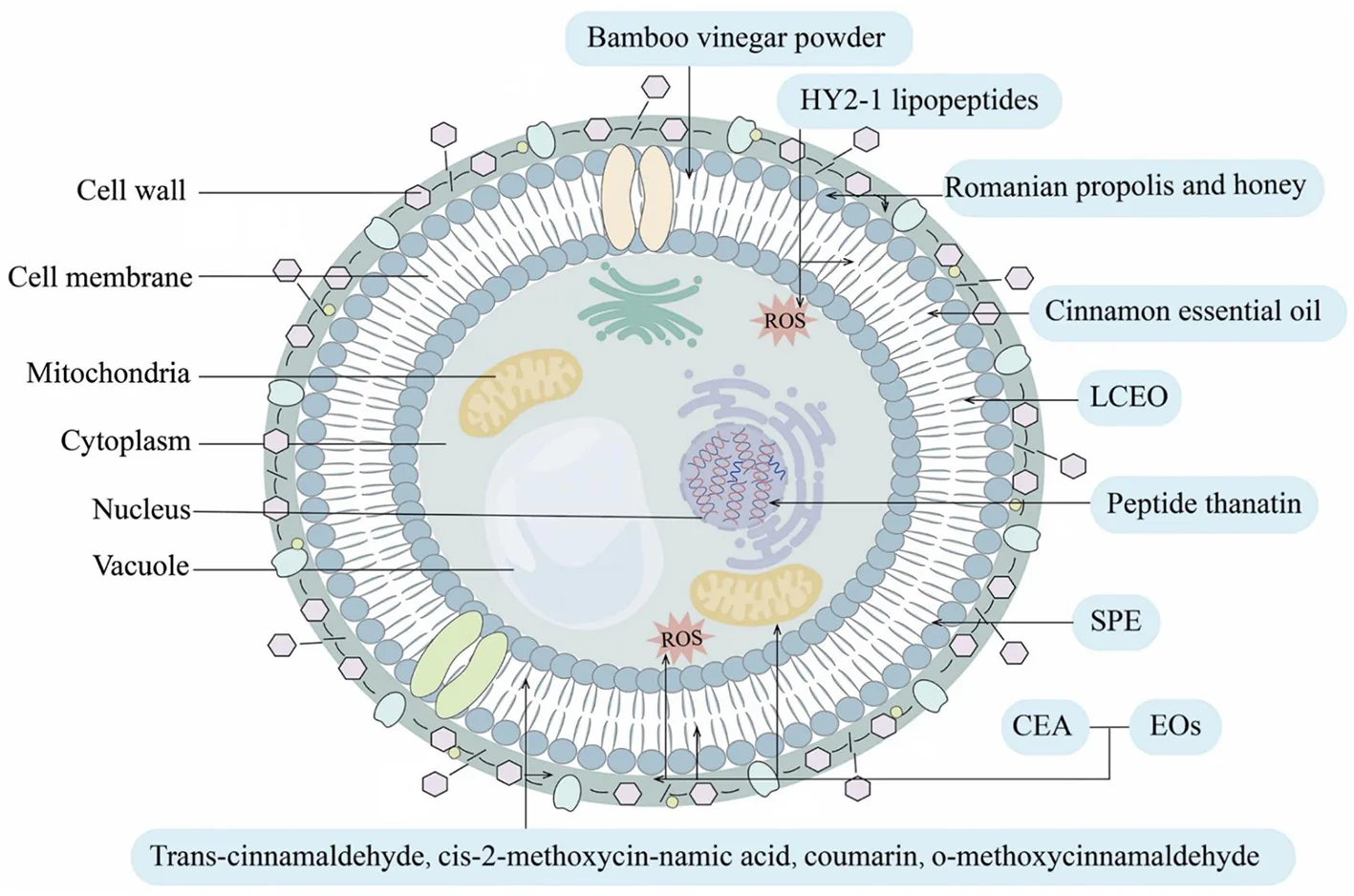
Potential effects of natural products against rare molds. ^†^CEA, citrus extract a; SPE, saffron petal extract; LCEO, *litsea cubeba* essential oil; EOs, essential oils.

Based on the analysis of case data, this article shows that the prognosis of rare mold infections remains poor. Rare molds, including *P. citrinum, P. chrysogenum, P. oxalicum, P. digitatum* and *P. variotii* pose a serious threat to immunocompromised patients. Currently, the global guideline for the diagnosis and management of rare mold infections recommends effective antifungal agents for rare mold infections, such as voriconazole, terbinafine, and liposomal amphotericin B. These antifungal agents mainly exert their effects by inhibiting ergosterol synthesis and damaging the fungal cell membrane. However, due to the increased drug resistance of rare molds and the significant side effects of antifungal agents, the treatment outcomes are often unsatisfactory. Especially for invasive deep mold infections, the prognosis for patients remains poor. Thus, it is evident that the development of novel antifungal agents is extremely urgent. Compared with other antifungal agents, the side effect risks of natural products may be lower, and they may also help to delay the emergence of fungal drug resistance (Sassi et al., 2022). According to published evidence, natural products have several significant advantages in combating rare mold infections, including multi-target activity, high efficacy, synergy, and natural origin. Through active exploration, researchers have discovered a series of natural products with unique mechanisms of action, which operate via targets distinct from those of conventional antifungal agents and thus offer new strategies to overcome drug resistance of rare molds. Natural products, such as trans-cinnamaldehyde, cis-2-methoxycinnamic acid, coumarin, and o-methoxycinnamaldehyde (Liu Q. et al., 2024), as well as citrus extract A (CEA) and essential oils (Eos) (Sassi et al., 2022), have been further suggested, via *in vitro* experiments including the microdilution method, to exert activity against *P. citrinum* by disrupting the fungal cell membrane and damaging the cell wall. Natural products, such as peptide thanatin (Feng et al., 2020) and saffron petal extract (SPE) (Naim et al., 2022), have been confirmed through *in vitro* and *in vivo* experiments to inhibit *P. digitatum* by suppressing RNA and protein synthesis, impairing cellular stress responses, disrupting the fungal cell membrane, and inhibiting fungal growth. Xin Xu et al. isolated and identified the strain bacillus amyloliquefaciens HY2-1 from the marine environment. Through *in vitro* experiments, it was confirmed that the HY2-1 lipopeptides mainly damage the integrity of fungal cell membranes and cause oxidative damage, thereby inhibiting *P. digitatum* (Liao et al., 2025). Litsea cubeba essential oil (LCEO) and cinnamon essential oil have been proven *in vitro* to inhibit *P. oxalicum* through multiple mechanisms, including disrupting cell membrane integrity, impairing mitochondrial function, triggering oxidative stress and reactive oxygen species (ROS) accumulation, suppressing spore germination, and reducing ergosterol synthesis (Yu et al., 2024; Liu W. et al., 2024). Romanian honey and propolis were found to exhibit antifungal activity against *P. chrysogenum* by the agar diffusion method. The diameter of the inhibition zone ranged from 7 to 12 millimeters in honey samples and from 15 to 28 millimeters in propolis samples. The honey was determined to be effective at a dilution as high as 1/16 (w/v), while the propolis remained effective at concentrations as low as 1.56 mg/mL for certain strains (Vicǎ et al., 2022). Yu-Pei Chen et al. confirmed through experiments that the Bamboo vinegar powder, processed from natural bamboo vinegar, contains the main active components. It disrupts the integrity of cell membranes through the key active component 4-methylbenzoic acid and interferes with the unique shikimate pathway of fungi, thereby exerting an anti-*P. variotii* activity (Chen et al., 2025). Zataria multiflora (ZEO) and Cinnamomum zeylanicum (CEO) vapor-phase essential oils exert antifungal activity against *P. citrinum* via physical volatile diffusion and chemical synergistic effects (Imaz et al., 2023) (Table 1, Figure 11).

**Table 1:** Natural products for the treatment of rare mold infections.

Species	Natural products^†^	Antifungal activity^‡^	Antifungal mechanism	Reference
*P. citrinum*	ZEO	MIC: 800 μL/L, Inhibition zone diameters: The CEO alone produced a 65.3 mm large antibacterial zone on the first day.	–	Imaz et al., 2023
	CEO	MIC: 400 μL/L		
	Trans-cinnamaldehyde	MIC = 0.3 mmol/L	Damaging the cell membrane, destroying the cell wall, and inhibiting energy metabolism.	Liu Q. et al., 2024
	Cis-2-methoxycin-namic acid	MIC = 0.44 mmol/L		
	Coumarin	MIC = 8.55 mmol/L		
	O-methoxycinnamaldehyde	MIC = 0.96 mmol/L		
	CEA	MIC = 156.25 ppm	Damaging the integrity of the cell membrane, inhibiting the synthesis of the cell wall, inducing oxidative stress, and causing mitochondrial damage.	Sassi et al., 2022
	EOs	MIC = 1250 ppm;		
*P. digitatum*	Peptide thanatin	• MIC = 2 μmol/L • MFC = 128 μmol/L	Inhibiting genetic information processing, disrupting amino acid metabolism, and inducing cellular stress.	Feng et al., 2020
	SPE	10% SPE: the complete inhibitation of mycelial growth and spore germination.	Damaging the integrity of the cell membrane.	Naim et al., 2022
	HY2-1 lipopeptides	• 2.4 mg/mL: delaying and inhibiting spore germination. • 2–8 mg/mL: forming a clearly inhibitory zone.	Damaging the integrity of the cell membrane and inducing oxidative damage.	Liao et al., 2025
*P. oxalicum*	LCEO	MIC = 2.24 g/L MFC = 4.48 g/L	Damaging the integrity of the cell membrane and causing oxidative stress.	Yu et al., 2024
	Cinnamon essential oil	MIC = 0.025% v/v MFC = 0.03% v/v ≥0.02%: completely inhibiting spore germination.	Damaging the integrity of the cell membrane.	Liu Q. et al., 2024
*P. chrysogenum*	Romanian honey	MIC = 1/16–1/32(w/v)	Damaging the cell membrane and cell wall.	Vicǎ et al., 2022
	Romanian propolis	MIC = 1.56–6.25 mg/mL		
*P. variotii*	Bamboo vinegar powder	• MIC = 2.5 mg/mL • 8-mg BVPE paper disc: forming a distinct antibacterial zone.	Damaging the integrity of the cell membrane and interfering with the unique shikimic acid metabolic pathway of fungi through key active component, 4-methylbenzoic acid.	Chen et al., 2025

† ZEO, zataria multiflora essential oils; CEO, cinnamomum zeylanicum essential oils; CEA, citrus extract a; SPE, saffron petal extract; LCEO, litsea cubeba essential oil; EOs, essential oils.

‡ MIC, minimum inhibitory concentration; MFC, minimum fungicidal concentration; BVPE, bamboo vinegar powder extract.

Due to the scarcity of rare mold species and limited relevant research, 10 natural products were found to have antifungal activity against 5 rare mold species. The literature has demonstrated that these natural products exert significant inhibitory or fungicidal effects on target molds both *in vitro* and in food models such as cheese and citrus. These substances are derived from plants, insects, and marine environments. Compared with traditional chemical disinfectants, they are generally considered environmentally friendly and carry a lower risk of inducing drug resistance. They are efficient, low-toxicity natural alternatives. Natural products summarized in these studies have shown promising effects against rare molds. Although multiple studies have explored their antifungal mechanisms, precise identification of molecular targets and molecular-level mechanistic validation remain insufficient, and *in vivo* animal experiments are still limited. Future research should focus on addressing these key shortcomings.

## 3 Discussion

A study once estimated that each year, over 6.55 million people are affected by fungal diseases that poses a direct threat to life, resulting in over 3.75 million deaths. Among them, approximately 2.55 million can be directly attributed to this fungal disease (Denning, 2024). Invasive fungal infections caused by molds other than *Aspergillus* are a significant cause of morbidity and mortality (Morrissey, 2025). As the number of patients requiring intensive care increases and the number of immunocompromised patients rises, infections caused by rare molds other than *Aspergillus spp*. or *Mucorales* are also on the rise (Hoenigl et al., 2021). These rare molds mainly originate from natural environments, such as soil, decaying plants, lakes, moldy citrus fruits and grains.

**Figure 12:**
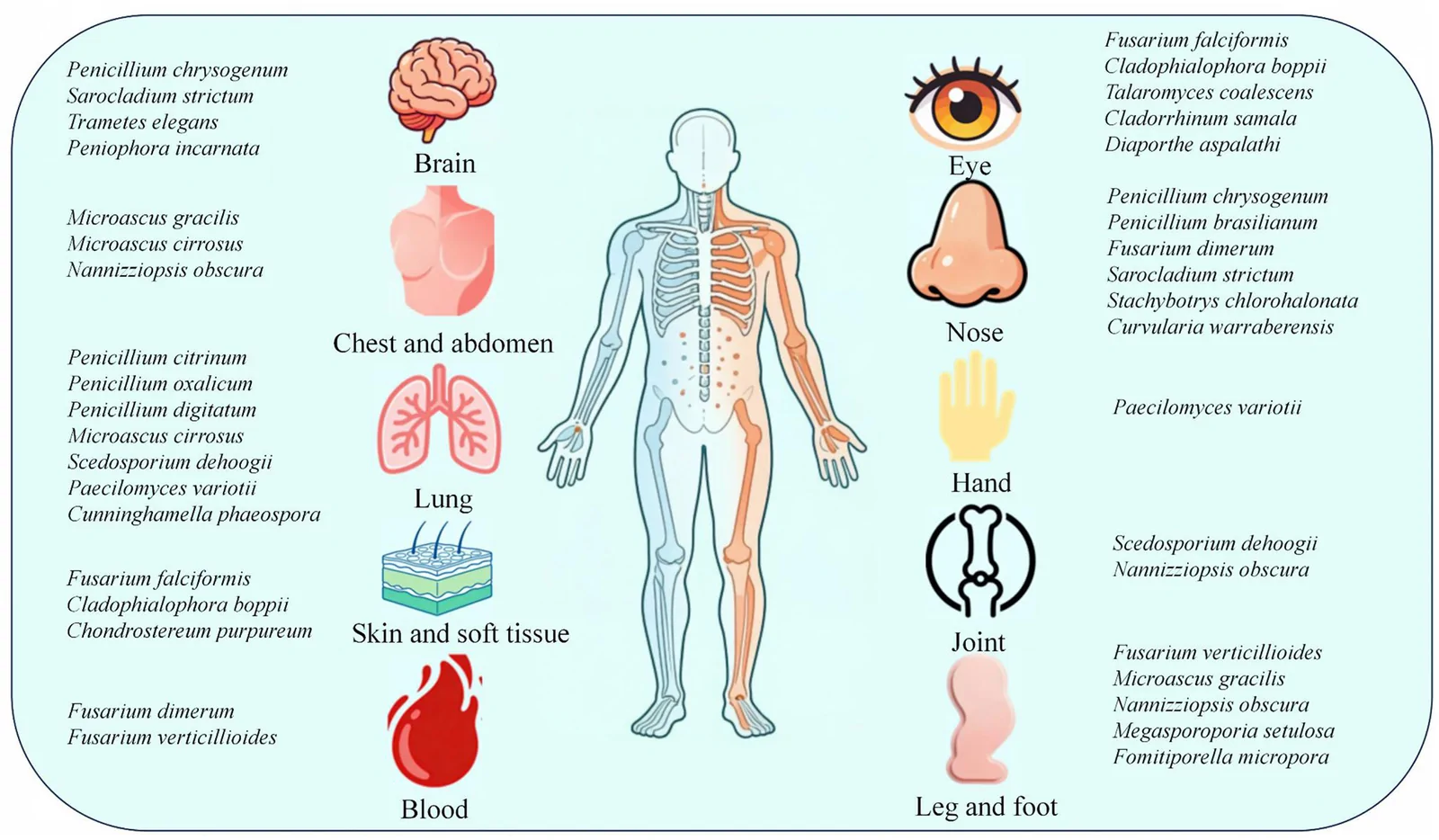
Anatomical distribution of rare molds in the human body.

In recent years, the number of invasive mold infection cases caused by rare molds has been increasing continuously, posing a major challenge. According to the clinical data summarized in this review, rare molds exhibit strong invasive capacity in humans. Most of these cases occur in individuals with multiple underlying diseases or compromised immune systems, while a few occur in individuals with normal immune systems. The clinical manifestations of rare mold infections show dual heterogeneity in susceptible populations and infection sites. As shown in Table 2, the cure rate of rare fungal infections is relatively high in patients with normal immune function, while it significantly decreases in individuals with compromised immune systems. From this, it can be inferred that the integrity of the immune system is the key factor determining whether rare mold infections can be controlled. Restoring the patient's immune function as soon as possible is crucial for controlling rare mold infections. As shown in Figure 12, the lungs are the most common site for rare mold infections, accounting for approximately one quarter (11/43, 26%) of all infection sites; the nose is the second most common site (6/43, 14%); the skin and soft tissue include fingernail, paratracheal abscess, shoulder, as well as rare mold infections in the eyes and limbs, each accounting for approximately 11% (5/43); the head and thoraco-abdominal region account for approximately 9% (4/43); blood, joints, and hands account for a relatively small proportion, with each site at roughly 4% (2/43). This indicates that these rare molds are highly invasive and can infect multiple parts of the human body, causing both superficial and deep infections. For rare mold infections, accurate identification of rare mold species is the primary prerequisite for targeted treatment. The development of mass spectrometry techniques (e.g., MALDI-TOF MS) and molecular sequencing technologies (e.g., DNA sequencing) has enabled the accurate identification of an increasing number of rare molds (Song et al., 2021). MALDI-TOF MS is an efficient but highly database-dependent rapid diagnostic technique (Al-Hatmi et al., 2015). With a complete database in place, the species-level identification rate is close to 90%, which is highly consistent with the molecular results (Song et al., 2021). DNA sequencing is the general term for all nucleic acid sequencing techniques and is the gold standard for mold identification (Visagie et al., 2014). Among them, ITS sequencing is used for precise species identification of molds, while mNGS is used as an unbiased high-throughput sequencing method to detect rare or non-cultivable pathogens (Zhao et al., 2024). DNA sequencing also includes PCR sequencing and β-tubulin gene sequencing. The case of *penicilliosis* described in this study lacked corresponding database entries, so it could not be accurately identified by MALDI-TOF MS and was finally confirmed as *P. brasilianum* via β-tubulin gene sequencing (Hirao et al., 2024). Therefore, when there is a high suspicion of rare mold infections, the combination of mass spectrometry and molecular sequencing techniques may be necessary for accurately identifying the species of mold. Furthermore, among the cases summarized in this article, several cases employed a combined approach of mass spectrometry and molecular techniques, or a combination of two different types of molecular techniques for identification (De Oliveira et al., 2023; Risum et al., 2022; Cui et al., 2021; Mascitti et al., 2022; Jarrin Jara et al., 2025; Matsuo et al., 2025). It can be seen that the identification method combining different types of technologies not only compensates for the limitation of MALDI-TOF MS in accurately identifying rare molds due to database insufficiency, but also leverages the high resolution and specificity of molecular identification techniques for the detection of rare molds. This suggests that for invasive rare mold infections, a combined identification strategy using multiple techniques can enhance the accuracy of identifying rare molds. The application of these identification techniques has enabled the earlier and more accurate diagnosis of numerous rare molds that were previously difficult to identify, significantly improving the clinical outcomes of patients.

**Table 2:** General information and description of cases of human infections with rare molds.

Species	Year of publication, country or region	Age/sex, underlying diseases^†^	Sources of clinical samples	Identification techniques	Antifungal susceptibility (MIC μg/mL)^§^	Antifungal treatments^‡^	Outcomes^¶^	Reference
*P. citrinum*	2020, China	67/M, T2DW	Lung	PCR sequencing	AMB: 2, VRC: >8	AMB: 10 mg/d-40 mg/d	Recovery	Zhao et al., 2020
	2021, India	60/M, multiple myeloma (stage II)	Lung	DNA sequencing	–	AMB (IV): 3–5 mg/kg/d	Recovery	Beena et al., 2021
*P. chrysogenum*	2022, Brazil	14/F	Brain	ITS + β-tubulin gene sequencing	–	VRC (IV): 300 mg q12h + AMB (IV): 1 mg/kg/d	Death^*^	De Oliveira et al., 2023
	2022, America	Adolescent/M, T-ALL	Nasal cavity	PCR sequencing	–	AMB (IV): 3–5 mg/kg/d	Recovery	Bhavsar et al., 2022
*P. oxalicum*	2024, China	56/F, T2DW	Lung	ITS sequencing	FLC: >256, AMB: 0.25, VRC: 0.38, ITR: 0.5	AMB (IV): 400 mg/d, 6 mg/kg/d	Recovery	Wang et al., 2024
*P. digitatum*	2022, Chile	20/F, pregnancy	Lung	β-tubulin gene sequencing	AMB: 3, VRC: 2, ITR: 4	ITR: 400 mg/d then 200 mg/d	Recovery	Iturrieta-González et al., 2022
	2024, China	56/M, chronic lung disease	Lung	mNGS	AMB: 2, VRC: 0.026, ITR: 0.125, MCF: ≤ 0.25	ITR (PO): 200 mg, bid	Recovery	Shi et al., 2024
	2026, China	66/M, EBV+DLBCL-NOS	Lung	mNGS	–	VRC	Death	Chen et al., 2026
*P. brasilianum*	2024, Japan	60/F, T2DW	Paranasal sinus	β-tubulin gene sequencing	AMB:2, FLC: >64, VRC: 4, ITR: 1, CPN: 0.25	AMP (IV: 250 mg/d, 5 mg/kg/d; VRC (PO): 300 mg bid, then 150 mg bid; ITR (PO): 200 mg bid then 200 mg qd	Death	Hirao et al., 2024
*F. dimerum*	2021, Saudi Arabia	38/F, short bowel syndrome, SARS-CoV-2 etc.	Blood	–	–	48–50 day: VRC (IV), The first day: 6 mg/kg q12h; next day: 4 mg/kg q12h; 51–59 day: AMB (IV), 5 mg/kg/d	Recovery	Alshaya et al., 2021
	2022, Denmark	49/F, AML	Paranasal sinus	ITS+18S rDNA sequencing	POS: >4, VRC: 4–8, ITR: >16, AMB: 0.5–1, TBF: 0.5–1, ISA: 16–>16	AMB (IV): 3 mg/kg/d (7 months); VRC (IV): 200–400 mg, bid; TBF (PO): 250 mg, qd; 500 mg, tid	Recovery	Risum et al., 2022
*F. falciformis*	2022, America	55/M, AML	Skin	DNA sequencing of the TEF and RPB2 genes	AMB: 0.5, VRC/ISA/POS: >16, CPN: >8, TBF: >2,	AMB 5 mg/kg/d	Hospice care	Jabr et al., 2022
	2025, America	54/M, seronegative rheumatoid arthritis	Cornea	–	AMB:1, VRC: >16, POS: >16, ISA: >16, NTM: 8, TBF: 2	L-AMB (IV): 5 mg/kg/d, add up 2.55 g + POS (PO): first 300 mg bid × 2d, then 300 mg qd;	Recovery	Larimore and Libertin, 2025
*F. verticillioides*	2022, Argentina	68/M, COVID-19 pneumonia	Blood	MALDI-TOF MS	AMB: 2, POS: 0.25, VRC: ≥8	Sodium deoxycholate AMB (IV): 1 mg/kg/d + VRC (IV): 400 mg/12qh, 200 mg/12qh	Death	Barberis et al., 2022
	2024, China	64/M, senile lower limb arterial occlusion	Left foot	Amplify the TEF1 gene and sequence it	MCF: >8, TBF: 0.5, AMB: 0.5, VRC: 2, ITR: 4	ITR (PO): 200 mg, bid	Recovery	Feng et al., 2024
*M. gracilis*	2020, America	65/M, immunosuppression after bilateral lung transplantation	Pleural effusion	DNA sequencing of the loci of D1/D2, TUB, and TEF.	AMB: 1, VOR: >16, POS: >16, ISA: 2, TBF: 2, MCF: 1	VRC (IV): 300 mg q12h; AMB (INH): 50 mg three times a week + AMP (IV): 2 g, q8h × 21 day	Death^*^	Ding et al., 2020
	2021, Sudan	50/F, left foot injury	Left foot	ITS amplification and sequencing	AMB: >4, 5-FC: >64, TBF: >16, CPN: >16	ITZ: 200 mg, bid	Recovery	Mhmoud et al., 2021
*M. cirrosus*	2021, China	72/F, bronchiectasis	Lung	28S rDNA PCR sequencing	–	VRC (PO): 200 mg bid + AMB (INH): 12.5 mg bid	Recovery	Liu et al., 2021
	2026, Thailand	59/M, end-stage renal disease	Abdomen	ITS sequencing	AMB: 8, FLC/ITR/VRC/ISA/POS/ CPN/TBF >16	AMB deoxycholate (IV): 1 mg/kg/d; VRC (PO): 6 mg/kg, bid, 4 mg/kg bid	Recovery	Tianprasertkij et al., 2026
*S. dehoogii*	2021, America	45/M	Joint	uPCR	–	VRC (PO): first 400 mg q12h, then 300 mg bid + TBF (PO): 250 mg qd	Recovery	Krauth et al., 2021
	2025, Japan	57/F, ALL	Lung	ITS sequencing	AMB: 16, VRC: 8, MCF: >16	MCF (IV): 50 mg/d; VRC (IV): 6 mg/kg bid, 3 mg/kg bid; L-AMB (IV): 3 mg/kg/d; VRC (PO): 200 mg bid; MCF (IV): 150 mg/d	Recovery	Yamazaki et al., 2025
*P. variotii*	2021, Brazil	48/F, T2DW	Left hand	–	–	ITR (PO): 200 mg/d; TBF (PO): 250 mg/d	Recovery	Criado et al., 2021
	2023, America	43/F, SLE	Lung	MALDI-TOF MS	AMB/ITR/POS: ≤ 0.03, VRC: 4	POS: 300 mg/d; MCF (IV): 150/mg/d	Recovery	Pechacek et al., 2023
	2024, China	56/F, T2DW	Lung	ITS sequencing	FCA: >256, AMB: 0.5, VRC: >32, ITR: 0.5	AMB (IV): 400 mg/d, 6 mg/kg/d	Recovery	Wang et al., 2024
*C. boppii*	2024, Japan	83/M	Cornea	DNA sequencing	MCF: ≤ 0.015, 5-FU: 0.5, AMB: 1, MCZ: >16, ITR/VRC: >8, FLC: >64, CPN: 8	ITR (PO): 200 mg/d	Recovery	Fukuoka et al., 2024
	2024, Japan	61/M	Fingernail	ITS sequencing	–	5% luliconazole for 8 weeks	Recovery	Fukuyama and Yaguchi, 2024
	2025, Netherlands	69/F, stage IV breast cancer	Skin	ITS1 and ITS4 sequencing	AMB: 1, ITR: 0.125, VRC: 0.25, POS: 0.063, ISA: 0.5, FLC: >64, TBF: 0.016	ITR (PO): 200 mg/d	Recovery	Cavens et al., 2025
*T. coalescens*	2023, Japan	77/M, chronic eye diseases	Eye	ITS sequencing	NTM: 2, AMB: 1, VRC: >8, FLC: >64, MCF: >16	5% azithromycin eye drops, once every hour	Recovery	Todokoro et al., 2023
*S. strictum*	2021, China	51/F, spontaneous rhinorrhea	Brain	mNGS + ITS sequencing	–	AMB (IV): 1 mg/d; 5-FU (PO): 6 g/d; VRC: 4.0 mg/kg, q12h; ITR (PO)	Deterioration	Cui et al., 2021
	2025, China	74/F	Nose	28S rRNA amplification and sequencing	–	VRC (PO): 200 mg bid	Recovery	Wang et al., 2025
	2026, China	42/F, sarcoidosis	Brain	mNGS	–	VRC (PO): 0.2 g, q12h + L-AMB (IV): 0.01 g/d−0.06 g/d	Death^*^	Huang et al., 2026
*N. obscura*	2022, France	56/M, kidney transplant	Joint	ITS + MALDI-TOF MS	VRC: 0.008, POS: 0.19, ISA: 0.064, ITR: 0.38, AMB: 0.125 MCF: 0.023	VRC (PO): 3 mg/kg bid	Recovery	Mascitti et al., 2022
	2023, Spain	45/F, kidney transplant	Breast	ITS sequencing	MCF: 0.03, ANI: 0.03, VRC: 0.01, ISA: 0.25, POS: 0.06, ITR: 0.25, AMB: 0.06, TBF: 0.5	ANI (IV): 100 mg/d	Recovery	Jiménez-García et al., 2023
	2024, America	58/F, kidney transplant	Left leg	Gene sequencing	–	VRC and AMB	Deterioration	Soyele et al., 2024
*C. purpureum*	2023, India	61/M	Abscess	DNA sequencing	–	VRC (PO): 400 mg, tid then 200 mg, tid	Recovery	Dutta and Ray, 2023
		2026, Thailand	65/F, Swelling on the right shoulder	Shoulder	ITS + D1/D2 sequencing	–	POS (PO): 6 months	Recovery	Kijacharoenchai et al., 2026
*S. chlorohalonata*	2021, America	23/M, ALL	Paranasal sinus	Gene sequencing	AMB: 0.06, MCF: 0.125, POS: 4, VRC: 2	ABLC and MIN	Death	Semis et al., 2021
*C. warraberensis*	2022, India	33/F, PTB	Paranasal sinus	ITS sequencing	AMB: 1, ITR: 0.25, POS: 0.125, VRC: 2, FLC: 32	L-AMB (IV): 5 mg/kg/d + ITR (IV/PO): 200 mg, q12h; 200 mg/d	Death^*^	Samaddar et al., 2023a
*C. samala*	2022, India	35/M	Cornea	PCR sequencing	–	5% nalidixic acid eye drops + 1% voriconazole eye drops	Recovery	Gupta et al., 2022
*M. setulosa*	2023, India	42/M	Left foot	DNA sequencing	AMB: 0.5, POS/VRC: 0.06, ITR: 0.25, FLC: ≥64, CPN: 16	Sodium Deoxycholate AMB:1 mg/kg/d, 2 weeks + ITR: 200 mg/d, 6 months	Recovery	Samaddar et al., 2023a
*F. micropora*	2025, America	69/M, heart transplantation	Right leg	ITS+28S rRNA sequencing	–	POS	Recovery	Jarrin Jara et al., 2025
*T. elegans* and *P. incarnata*	2025, Japan	27/M, AIDS	Sputum	ITS+28S rDNA sequencing	–	L-AMB (IV): 3 mg/kg/d; VRC (IV): 6 mg/kg, q12h	Recovery	Matsuo et al., 2025
*D. aspalathi*	2025, China	50/F	Cornea	mNGS	–	ITR (PO): 200 mg/d; VRC: 1% of the area, once per hour	Recovery	Huang et al., 2025
*C. phaeospora*	2025, Japan	71/M, T2DW/ALL	Lung	ITS + D1/D2 sequencing	AMB:4, MCF/CPN/ITR/VRC/ POS/ISA: >8	ISA (IV): 200 mg, qd; L-AMB (IV): 5 mg/kg/d	Death^*^	Shimada et al., 2025

† F, female; M, male; SARS-CoV-2, severe acute respiratory syndrome coronavirus 2; T2DW, type 2 diabetes; EBV+DLBCL-NOS, Epstein–Barr virus-positive diffuse large B-cell lymphoma, not otherwise specified; COVID-19, coronavirus disease 2019; T-ALL, T-cell acute lymphoblastic leukemia; AML, acute myeloid leukemia; AIDS, acquired immunodeficiency syndrome; SLE, systemic lupus erythematosu; PTB, pulmonary tuberculosis.

‡ AMB, amphotericin B; L-AMB, liposomal amphotericin B; ABLC, amphotericin B lipid complex; VRC, voriconazole; FLC, fluconazole; ITR, itraconazole; MCF, micafungin; CPN, caspofungin; POS, posaconazole; TBF, terbinafine; ISA, isavuconazole; NTM, natamycin; 5-FC, flucytosine; MCZ, miconazole; ANI, anidulafungin.

§ –, Not mentioned.

¶* Death was caused by rare mold infections.

In 2021, the European Confederation of Medical Mycology in cooperation with the International Society for Human and Animal Mycology and the American Society for Microbiology issued the global guideline for the diagnosis and management of rare mold infections (Hoenigl et al., 2021). This guideline recommends first-line therapeutic agents for rare mold infections, mainly traditional antifungals such as amphotericin B, voriconazole and terbinafine. Based on the case data summarized in this article, drug susceptibility and the therapeutic effects of antifungal agents are basically consistent. Most infections improve significantly after treatment with highly susceptible antifungal agents recommended by the global guideline for the diagnosis and management of rare mold infections. Some antifungal agents with moderate susceptibility can also serve as long-term oral regimens for patients with intact immune function, achieving satisfactory clinical outcomes (Feng et al., 2024). However, there are some cases suggesting that there is a significant difference between the antifungal susceptibility testing results and the clinical efficacy. *In vitro* drug resistance does not necessarily mean clinical ineffectiveness (Tianprasertkij et al., 2026). In the aggregated case reports, some patients did not undergo antifungal susceptibility testing. Despite empirical antifungal administration, patients eventually died due to rapidly progressive infection (De Oliveira et al., 2023). This indicates that conducting timely antifungal susceptibility testing and selecting appropriate antifungal agents may potentially increase the cure rate of rare mold infections. *Microascus spp., Cladophialophora spp., Sarocladium spp., Nannizziopsis spp*., and *Chondrostereum spp*., which are mentioned in this article have not been included in the global guideline for the diagnosis and management of rare mold infections. In addition, *M. gracilis, S. strictum, N. obscura, C. purpureum* as well as the rare molds mentioned in Section 2.11, have been reported to cause human infections for the first time since 2020 and have also not been included in the global guideline for the diagnosis and management of rare mold infections. For these rare molds not included in the global guideline for the diagnosis and management of rare mold infections, although there are no recommended first-line treatment drugs for their infections, selecting medications based on antifungal susceptibility testing and combining this approach with aggressive surgical debridement can still achieve satisfactory therapeutic outcomes. When clinicians encounter such rare mold infection cases and lack relevant antifungal recommendations, they should draw on the antifungal treatment experience summarized in this article to implement individualized therapy.

It is worth noting that although traditional antifungal agents are relatively effective in treating rare mold infections, there are still many cases of rare mold infections that result in death due to drug resistance or ineffective antifungal treatment (Shimada et al., 2025). Therefore, for rare mold infections, it is of great urgency to accelerate the exploration of potential therapeutic drugs. Natural products play a vital role in the research and development of agents against rare mold infections, acting via unique targets including fungal cell walls, membrane structures, and metabolic pathways, which differ from the targets of conventional antifungal drugs. For instance, cinnamon essential oil can inhibit *P. oxalicum* by disrupting cell membrane integrity (Liu Q. et al., 2024). Some natural products have demonstrated *in vitro* activity against drug-resistant molds. This indicates that they can provide some new ideas for the development of antifungal agents to prevent and control these rare mold infections. These findings provide a significant scientific basis for developing novel drugs against rare mold infections and improving patient prognosis, while also opening new avenues for the clinical treatment of such infections.

## 4 Conclusion

Currently, the number of cases of rare mold infections is increasing, and new human pathogenic mold species are being discovered, posing a serious threat to immunocompromised hosts. This review systematically summarizes the progress in the epidemiology, diagnosis, and treatment of rare mold infections from January 2020 to February 2026. The comprehensive analysis indicates that these rare mold infections mainly affect immunocompromised individuals and often present as severe clinical syndromes such as meningoencephalitis, disseminated or invasive pulmonary infections. In immunocompetent individuals, environmental mold spores may enter the subcutaneous tissue through trauma, particularly penetrating wounds or contusions, thereby triggering invasive mold infections.

The core problems of current rare mold infections include delayed diagnosis, limited treatment options, and a lack of unified diagnostic and therapeutic standards. In terms of diagnosis, the combined application of mass spectrometry and molecular techniques, or the use of multiple molecular assays in tandem, enables accurate identification of morphologically indistinguishable rare mold species. The effect may be superior to that of a single identification method. In terms of treatment, although the antifungal agents recommended by the global guideline for the diagnosis and management of rare mold infections are usually effective, drug selection can still be further optimized in clinical practice. For instance, combination therapy based on liposomal amphotericin B plus azole agents such as posaconazole can help control patients' *P. chrysogenum* infection via long-term maintenance treatment. Additionally, there are cases where antifungal therapy based on antifungal susceptibility testing, rather than following the global guideline for the diagnosis and management of rare mold infections, has also achieved favorable outcomes. For rare mold infections not covered by the global guideline for the diagnosis and management of rare mold infections, favorable therapeutic outcomes can be achieved through antifungal susceptibility testing, empirical antifungal therapy, and surgical intervention. Finally, with advancing research on natural products, novel strategies for drug development may be provided for currently untreatable rare mold infections in the future.
